# FKBP51 mediates resilience to inflammation-induced anxiety through regulation of glutamic acid decarboxylase 65 expression in mouse hippocampus

**DOI:** 10.1186/s12974-022-02517-8

**Published:** 2022-06-15

**Authors:** Yu-Ling Gan, Chen-Yu Wang, Rong-Heng He, Pei-Chien Hsu, Hsin-Hsien Yeh, Tsung-Han Hsieh, Hui-Ching Lin, Ming-Yen Cheng, Chung-Jiuan Jeng, Ming-Chyi Huang, Yi-Hsuan Lee

**Affiliations:** 1 Department and Institute of Physiology, College of Medicine, National Yang Ming Chiao Tung University, 155 Sec. 2, Linong Street, Taipei, 112 Taiwan; 2 Brain Research Center, National Yang Ming Chiao Tung University, 155 Sec. 2, Linong Street, Taipei, 112 Taiwan; 3 Department of Mathematics, Hong Kong Baptist University, 224 Waterloo Road, Kowloon Tsai, Hong Kong, China; 4 Department and Institute of Anatomy and Cell Biology, College of Medicine, National Yang Ming Chiao Tung University, 155 Sec. 2, Linong Street, Taipei, 112 Taiwan; 5 Department of Psychiatry, Taipei City Psychiatric Center, Taipei City Hospital, 309 Song-De Street, Taipei, 110 Taiwan; 6 Department of Psychiatry, School of Medicine, College of Medicine, Taipei Medical University, 250 Wu-Xing Street, Taipei, 110 Taiwan; 7 Psychiatric Research Center, Taipei Medical University Hospital, 252 Wu-Xing Street,, Taipei, 110 Taiwan

**Keywords:** FK506-binding protein 51 (FKBP51), Inflammation, Anxiety, Resilience, Glucocorticoid receptor, Glutamic acid decarboxylase 65 (GAD65), GABA, Ventral hippocampus

## Abstract

**Background:**

Inflammation is a potential risk factor of mental disturbance. *FKBP5* that encodes FK506-binding protein 51 (FKBP51), a negative cochaperone of glucocorticoid receptor (GR), is a stress-inducible gene and has been linked to psychiatric disorders. Yet, the role of FKBP51 in the inflammatory stress-associated mental disturbance remained unclear.

**Methods:**

*Fkbp5*-deficient (*Fkbp5*-KO) mice were used to study inflammatory stress by a single intraperitoneal injection of lipopolysaccharide (LPS). The anxiety-like behaviors, neuroimaging, immunofluorescence staining, immunohistochemistry, protein and mRNA expression analysis of inflammation- and neurotransmission-related mediators were evaluated. A dexamethasone drinking model was also applied to examine the effect of *Fkbp5*-KO in glucocorticoid-induced stress.

**Results:**

LPS administration induced FKBP51 elevation in the liver and hippocampus accompanied with transient sickness. Notably, *Fkbp5*-KO but not wild-type (WT) mice showed anxiety-like behaviors 7 days after LPS injection (LPS-D7). LPS challenge rapidly increased peripheral and central immune responses and hippocampal microglial activation followed by a delayed GR upregulation on LPS-D7, and these effects were attenuated in *Fkbp5*-KO mice. Whole-brain [^18^F]-FEPPA neuroimaging, which target translocator protein (TSPO) to indicate neuroinflammation, showed that *Fkbp5*-KO reduced LPS-induced neuroinflammation in various brain regions including hippocampus. Interestingly, LPS elevated glutamic acid decarboxylase 65 (GAD65), the membrane-associated GABA-synthesizing enzyme, in the hippocampus of WT but not *Fkbp5*-KO mice on LPS-D7. This FKBP51-dependent GAD65 upregulation was observed in the ventral hippocampal CA1 accompanied by the reduction of c-Fos-indicated neuronal activity, whereas both GAD65 and neuronal activity were reduced in dorsal CA1 in a FKBP51-independent manner. GC-induced anxiety was also examined, which was attenuated in *Fkbp5*-KO and hippocampal GAD65 expression was unaffected.

**Conclusions:**

These results suggest that FKBP51/*FKBP5* is involved in the systemic inflammation-induced neuroinflammation and hippocampal GR activation, which may contribute to the enhancement of GAD65 expression for GABA synthesis in the ventral hippocampus, thereby facilitating resilience to inflammation-induced anxiety.

**Supplementary Information:**

The online version contains supplementary material available at 10.1186/s12974-022-02517-8.

## Background

The FK506-binding protein 51 (FKBP51), a member of the immunophilin superfamily encoded by *Fkpb5* gene, is a heat shock protein 90 cochaperone that interacts with the glucocorticoid receptor (GR) present in the cytosol to form a GR complex [1]. Activated GR translocates to the nucleus and binds to the glucocorticoid response element (GRE) on the *Fkbp5* promoter to drive gene transcription [1]. FKBP51 functions to reduce the affinity of the GR to glucocorticoid (GC) and the subsequent nuclear translocation of the GR complex, resulting in the negative feedback control of GR sensitivity [2]. This molecular network normalizes the hypothalamic–pituitary–adrenal (HPA) axis reactivity and ultimately constitutes an adaptive reaction to stress. However, genetic variations of *Fkbp5* that results in excessive FKBP51 expression has been linked to the pathogenesis of psychiatric disorders due to the increase of vulnerability to stress-related anxiety and depression [3–6]. While accumulating studies focused on the beneficial effect of FKBP51 inhibition in ameliorating stress-induced mental disorders [7, 8], the effect of FKBP51 hypofunction on brain health remained poorly understood.

A crucial function of endogenous GC is to protect the host from the detrimental effects of inflammatory immune responses [9, 10]. Impaired GR/HPA axis signaling could enhance inflammatory susceptibility, thereby contributing to various physical and psychological diseases [11]. Moreover, proinflammatory cytokines can alternatively affect the GR/HPA axis response, thus aggravating inflammation and exacerbating cytokine activity [12]. Considering that the GR is subjected to inflammatory responses and that its sensitivity is affected by cytokine release, the FKBP51-mediated regulation of GR activity can provide the basis for investigating the role of FKBP51 in inflammation-related conditions [13]. An in vitro study reported that FKBP51 modulated the innate immune response [14] and induced the expression of interferon and proinflammatory cytokines [15]. The silencing of *Fkbp5* resulted in decreased cytokine and chemokine secretion, indicating that attenuated FKBP51 expression exerts an anti-inflammatory effect [16]. A human study reported similar findings of an association between higher FKBP51 expression and an enhanced proinflammatory profile [17]. In addition, upregulated FKBP51 expression was observed in the bone marrow-derived mononuclear cells of patients with rheumatoid arthritis [18] and the sputum cells of patients with chronic obstructive pulmonary disease [19], indicating a role of FKBP51 in inflammation. On the basis of the relationship of FKBP51 with neuroendocrine and immune systems, the effect of immune challenges on stress-related behaviors and the underlying mechanisms should be examined.

Studies have indicated that either the peripheral inflammatory process secondary to physical illnesses or immunotherapy (particularly interferon-α) can cause depression and anxiety [20, 21]. Furthermore, patients with depressive or anxiety disorders exhibited increased levels of cytokines such as interleukin-6 (IL-6) and tumor necrosis factor-α (TNF-α) [22, 23]. These observations suggest a relationship between inflammation and emotional disturbances. Systemic inflammation caused by the administration of lipopolysaccharide (LPS), an endotoxin present in the outer membrane of Gram-negative bacteria, can stimulate innate immunity and generate various features of the acute inflammatory responses [24]. LPS-induced peripheral inflammation would induce microglial activation and neuroinflammation, either by blood–brain barrier (BBB) leakage to allow immune cell infiltration into the brain, or by transport of proinflammatory mediators into the brain via circumventricular organs [25, 26]. It has been reported that the elevation of proinflammatory cytokines in the brain, such as TNF-α, IL-1β, and IL-6, causes systemic inflammation-associated mood disorders and cognitive impairment [27, 28]. Peripheral LPS challenge-triggered depressive- or anxiety-like behaviors examined in the animal models were often at acute phase when the animals were in sickness, including fever, body weight loss, and decreased food intake, etc. [29, 30]. Yet, whether the acute inflammation has long-lasting impact on the behavioral responses after recovery from the sickness was less studied. Besides, mechanisms underlying the role of FKBP51 in the inflammation-associated stress adaptation and behavioral responses remained unclear.

A functional imbalance between excitatory and inhibitory synaptic transmission in the brain is associated with the pathogenesis of anxiety disorders [31]. Although glutamate is the major excitatory neurotransmitter in the central nervous system (CNS), γ-amino butyric acid (GABA) is the principal inhibitory neurotransmitter synthesized through glutamic acid decarboxylase 65 (GAD65) or GAD67 in the CNS and is considerably involved in the control of emotion, cognition, and behavior [32]. GABAergic activity might be closely bound to immune processes and signals and implicated in the pathogenic mechanisms of anxiety and depression [33]. The upregulation of cytokines, such as TNF-α, secondary to inflammation may result in the dysregulation of GABAergic neurotransmission [34]. However, studies evaluating the role of FKBP51 in the regulation of the GABAergic system following peripheral inflammation are scant.

Inflammatory reactions can affect neuroendocrine function, neurotransmitter metabolism, regional brain activity, and, eventually, behavior [35]. In the present study, by using *Fkbp5* knockout mice, we evaluated the regulatory role of FKBP51 in stress-related anxiety-like behavior after an LPS challenge. Hippocampus is a critical brain region in regulating stress responses, emotional processing, and motivated behavior, and the CA1 neurons in the ventral hippocampus modulate anxiety-related behaviors [36–38]. We examined whether and how FKBP51 mediates GABAergic neurotransmission in the hippocampus and its relevance to the pertinent behavior phenotype following LPS-induced stress responses. Our results provide crucial insight into the molecular pathways of the neuroendocrine-immune perturbation underlying emotional disturbances.

## Methods

### Animals

Twelve-week-old male C57BL/6JNarl (wild type [WT]) and conventional *Fkbp5* knockout (*Fkbp5*^*tm1Dvds*^/J [*Fkbp5*-KO]) mice were used. *Fkbp5*-KO mice were purchased from Jackson Laboratory (Bar Harbor, ME, USA) and were backcrossed and bred in the Laboratory Animal Center of National Yang Ming Chiao Tung University, Taipei, Taiwan. The mice were fed a chow diet and kept in a 12-h light/dark cycle at 25 ± 2 °C. All animal experiments were reviewed and approved by the Institutional Animal Care and Use Committee (IACUC) of National Yang Ming Chiao Tung University (IACUC number: 1050303, 1071001, and 1090110).

### LPS administration to induce transient peripheral inflammation

A single intraperitoneal injection of LPS (3 mg/kg of body weight; *Escherichia coli* O55:B5, Sigma-Aldrich, St. Louis, MO, USA) or vehicle (saline, SAL), which can induce neuroinflammation [39], was administered to 12-week-old male WT and *Fkbp5*-KO mice. The sickness behavior of all animals was assessed every day by measuring changes in their body weight and food intake. To determine peripheral and central inflammatory responses, the mice were killed on day 1 or day 7 after the LPS injection, and their liver and hippocampus were harvested for the mRNA analysis of TNF-α expression. Behavioral tests were conducted in LPS-injected mice on day 7 after the LPS injection. Subsequently, intracardial perfusion with phosphate-buffered saline (PBS) was performed to obtain brain tissues for the aforementioned biochemical and histological examinations.

### Behavioral assessment

Anxiety-related behaviors were assessed using the open-field test (OFT) and elevated plus maze (EPM) test [40]. In addition, we conducted a sucrose preference test to assess anhedonia. The traces of mouse movement during the OFT and EPM were recorded using Smart software (Panlab, S.L.U., Barcelona, Spain). The OFT was performed to examine spontaneous locomotor activity and anxiolytic behavior in an open-field maze, which was a 26 × 26-cm field with 30-cm-high walls. At the start of the trial, a mouse was placed in the center of the field and allowed to freely explore the field for 5 min. Spontaneous locomotor activity was calculated as the total distance traveled by the mouse in 5 min. The time spent by the mouse in the central zone during the 5-min recording period served as an index of the anxiolytic response. The EPM was performed to determine the anxiety-like behavior. The EPM apparatus comprised two open arms and two closed arms (30 × 5 cm) perpendicular to each other with the platform elevated 50 cm from the floor. At the start of the trial, a mouse was placed in the center of the apparatus and allowed to explore the maze for 5 min. Choice behavior was observed for 5 min, and the time spent in each arm was recorded. The time spent in the open arm during the 5-min recording period served as an index of the anxiolytic response, and the total distance traveled by the mouse represented locomotor activity in the EPM apparatus. The sucrose preference test was performed to examine depression-like behavior (anhedonia), and we used the test procedure described previously with some modifications [41]. The animals were not deprived of food or water before the test. For habituation, at a day prior to testing, the animals were allowed to drink a 2.5% sucrose solution for 2 h. After the completion of habituation, the sucrose solution was replaced with tap water for 22 h. For the sucrose preference test, each animal was transferred to a new cage with a clean bed. Subsequently, a bottle containing tap water and another bottle containing 2.5% sucrose solution were placed in each cage, and the animal was allowed to freely drink for 12 h. During the test, the place of bottles was exchanged after 6 h. Sucrose preference was calculated as the percentage of the consumption of sucrose divided by the total consumption of fluid intake.

### Quantitative real-time polymerase chain reaction

The total RNA of the mouse livers and hippocampus was extracted using TRIzol reagent (Invitrogen, Carlsbad, CA, USA) following the manufacturer’s instructions. cDNA was obtained through the reverse transcription of mRNA by using a high-capacity reverse transcription kit (Thermo Fisher Scientific, Waltham, MA, USA). Quantitative real-time polymerase chain reaction (qRT-PCR) was performed using an ABI StepOnePlus real-time PCR system by using the mouse primers listed in Additional file 1: Table S1. The endogenous 18s rRNA gene served as an internal control. The relative gene expression was determined using the ΔΔCt method, where Ct represented the threshold cycle.

### Western blot analysis

Tissue lysates were harvested and prepared by homogenizing the tissue in lysis buffer (50 mM Tris–HCl, 150 mM NaCl, 1% Triton X-100, 0.5% SDS, pH 7.4) supplemented with protease inhibitor cocktail I (Roche Applied Science, Penzberg, Germany), 1 mM Na_3_VO_4_, and phosphatase inhibitor cocktail (Sigma-Aldrich). Total proteins were electrophoresed and transferred onto a polyvinylidene fluoride (PVDF) membrane. The PVDF membrane was blocked with 5% skimmed milk dissolved in PBS with 0.05% Tween-20 and probed using the following primary antibodies:rabbit anti-FKBP51 (1:1000, Cell Signaling Technology, Danvers, MA, USA), rabbit anti-GR (1:1000, Santa Cruz, Dallas, Texas, USA), rabbit anti-NR2A (1:1000, Millipore, Bedford, MA, USA), rabbit anti-NR2B (1:1000, Millipore), mouse anti-GAD65 (1:1000, Proteintech, Rosemont, IL, USA), rabbit anti-GAD67 (1:1000, Abcam, Cambridge, UK), rabbit anti-GABA_A_R α1 (1:1000, Synaptic System, Goettingen, Germany), and rabbit anti-GAPDH (1:5000, GeneTex, Irvine, CA, USA). Furthermore, the probed membranes were incubated with horseradish peroxidase-conjugated secondary antibodies (Jackson ImmunoResearch, West Grove, PA, USA), and the protein bands were visualized using the Western Lightning Plus-ECL Enhanced Chemiluminescence Substrate (PerkinElmer, Waltham, MA, USA). The intensity of each protein band was analyzed using Image J software (National institutes of Health, Bethesda, MD, USA) and normalized to the intensity of the internal control GAPDH.

### Measurement of spleen weights and complete blood counts

For assessment of peripheral inflammatory responses, the spleen weights and complete blood counts were analyzed. The spleens were carefully removed without fat or connective tissue, and weighed using a microbalance. Peripheral blood samples were collected from the submandibular vein into BD Microtainer® blood collection tubes with the anticoagulant K_2_EDTA (BD, Franklin Lakes, NJ, USA) and analyzed on a complete blood counter (XT-1800i, Sysmex Europe, Norderstedt, Germany).

### Immunofluorescence staining and immunohistochemistry

Immunofluorescence staining was performed as previously described [42]. Briefly, mouse brain coronal cryosections (30-μm thickness) were permeabilized using PBS with 0.05% Tween-20, and then immunostained with rabbit anti-Iba-1 (Wako, Tokyo, Japan), mouse anti-GR (Invitrogen), and mouse anti-GAD65 (Abcam) antibodies diluted in PBS with 0.1% Tween-20, followed by incubation with an Alexa Fluor-conjugated secondary antibody (Invitrogen) and nuclear staining with 4′,6′-diamidino-2-phenylindole dihydrochloride (DAPI) in the mounting medium (Vector Laboratories, Burlingame, CA, USA). Fluorescent images were acquired using a fluorescence microscope (Leica DM6000B) or confocal laser scanning microscope (Olympus FV1000) and quantified using the MetaMorph version 7.7.0.0 software (Molecular Devices). The immunoreactive area of the Iba-1 expression was calculated as the ratio of the Iba-1 immunoreactive area to the area of the respective microscopic regions examined in stratum pyramidale (SP) and stratum radiatum (SR) of the hippocampus. Quantification of the cell number of nuclear GR-positive cells, the images of GR and DAPI staining were analyzed by the “Multi Wavelength Cell Scoring” tool of the MetaMorph to determine the percentage of GR-DAPI overlapping signals in the total number of DAPI-positive cells. The immunoreactive area of the GAD65 expression was calculated as the ratio of the GAD65 immunoreactive area to the number of total DAPI-positive cells in SP layer of dorsal CA1 and ventral CA1 subregion of hippocampus and central nucleus of the amygdala (CeA), and then normalized with saline group. For diaminobenzidine (DAB) staining, mouse brain cryosections were incubated with the mouse anti-c-Fos antibody (Abcam), followed by incubation with a biotinylated secondary antibody (Vector Laboratories). Subsequently, the sections were incubated with Vectastain Elite ABC reagent (Vector Laboratories) for 30 min and developed using a DAB peroxidase substrate kit (Vector Laboratories). Neuronal activity was indicated by the number of the c-Fos-positive neurons of specific regions. Three brain sections per animal were performed for immunofluorescence staining and immunohistochemistry. Three to five animals per group were analyzed and quantified as indicated in the figure legends.

### In vivo* animal 7-T positron emission tomography/magnetic resonance imaging and quantification for neuroinflammation*

For quantification of neuroinflammation, we used [^18^F]-*N*-(2-(2-fluoroethoxy)benzyl)-*N*-(4-phenoxypyridin-3-yl) acetamide ([^18^F]-FEPPA) positron emission tomography (PET)/magnetic resonance imaging (MRI) as previously described [43]. [^18^F]-FEPPA is a high affinity radioligand for the 18-kDa translocator protein (TSPO) expressed in the outer membrane of the mitochondria, used for detecting glial activation and neuroinflammation in PET [44, 45]. Briefly, mice anesthetized using a mixture of 2% isoflurane and 98% oxygen were injected with approximately 300–500 μCi/150 μL of [^18^F]-FEPPA in the tail vein. We used the two-dimensional mode on a 7-T PET/MRI system (Bruker, Germany) to acquire dynamic PET/MRI. The post-acquisition frame rebinning generated 18 frames (15 × 60 s and 3 × 600 s) of increasing length. Reconstructed images using an ordered subset expectation maximization (OSEM2D) algorithm was 0.86 mm transaxially with a slice thickness of 0.77 mm, using 16 subsets and 4 iterations yielded a spatial resolution of 1.5 mm at full width at half maximum at the center of the field of view. CT imaging data were corrected for attenuation by using the manufacturer’s software. Image processing was performed using PMOD3.7 software (PMOD Technologies, Zurich, Switzerland). Quantitative PET imaging was performed by examining the binding potential of [^18^F]-FEPPA using Logan graphical analysis.

### GC-induced stress model

To investigate the role of FKBP51 in the GC-induced anxiety, we used free drinking of dexamethasone (DEX, Sigma-Aldrich) to avoid handling- or injection-induced stress. DEX was dissolved in tap water (0.016 mg/mL) and provided the mice free drinking access for 7 days. After the treatment, behavior tests and biochemical analysis were performed to confirm that GC-induced HPA axis activation and anxiety-like behaviors were induced successfully.

### Statistical analysis

All statistical analyses were performed using GraphPad Prism 6 software (GraphPad Software, San Diego, CA, USA). Data are expressed as the mean ± standard error of the mean (SEM). Two-way analysis of variance (ANOVA) was performed, followed by a multiple-comparison Tukey post hoc test for between-group comparisons. Unpaired Student’s *t* test was used to compare between two groups for immunofluorescence and immunohistochemistry findings. The levels of statistical significance were *p < 0.05, **p < 0.01, ***p < 0.001.

## Results

### *Fkbp5* deficiency enhanced LPS-induced anxiogenic response

**Fig. 1:**
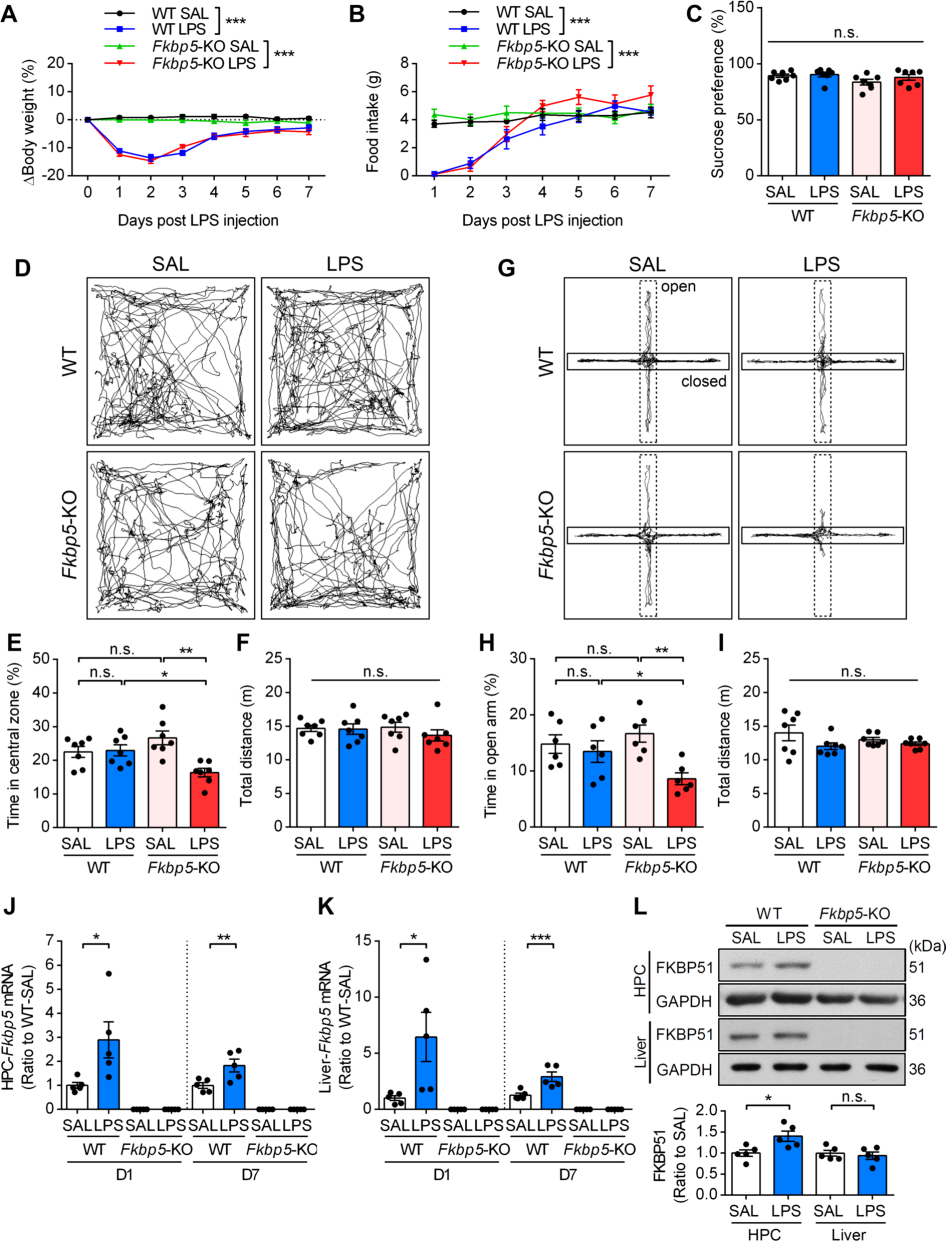
*Fkbp5* deletion increases sensitivity to LPS-induced anxiety responses. C57BL/6J (WT) and *Fkbp5*-KO mice were subjected to a single intraperitoneal injection of saline (SAL) or LPS (3 mg/kg of body weight). **A**, **B** The mean body weight (n = 7 mice per group) and food intake (n = 5 mice per group) of mice were tracked every day for 7 days to assess sickness behavior. Data are expressed as the mean ± SEM. ***p < 0.001 for the comparison between two groups in a two-way ANOVA. **C** The sucrose preference test was performed to assess the anhedonia-like phenotype. Representative traces (**D**) and the time spent in the central zone (**E**) of the open-field test (OFT). Spontaneous locomotor activity was expressed as the total distance traveled in the OFT (**F**). Representative traces (**G**), time spent in the open arms (**H**), and total distance traveled (**I**) in the elevated plus maze (EPM). *n* = 7 mice per group. **J**, **K** qRT-PCR analysis of both hippocampal (HPC) and liver *Fkbp5* expression at days 1 and 7 after SAL or LPS injection (n = 5 mice per group). *p < 0.05, **p < 0.01, and ***p < 0.001 for the comparison between two groups in a two-way ANOVA followed by the Tukey post hoc test. **L** Western blotting indicated that LPS increased FKBP51 protein expression at day 7 in the HPC (*n* = 5 mice per group). The relative band intensities were normalized to GAPDH levels. *p < 0.05 for the comparison between two groups in an unpaired Student’s *t* test

To examine the role of FKBP51 in inflammation-induced anxiety, we treated 12-week-old WT and *Fkbp5*-KO mice with a single bolus intraperitoneal injection of the bacterial endotoxin LPS (3 mg/kg of body weight). LPS-induced sickness was examined by measuring body weight and food intake from days 1 to 7 after saline or LPS injection. The results showed that LPS injection induced transient sickness (i.e., reduction in body weight and food intake) in the first 3 days in both WT and *Fkbp5*-KO mice, and the body weight and food intake of these mice recovered from days 5 to 7 to a level similar to those of their respective saline-injected control mice (Fig. 1A, B). LPS-induced sickness did not significantly differ between WT and *Fkbp5-*KO mice. Subsequently, we evaluated the levels of anhedonia and anxiety in mice on day 7 after saline or LPS injection (LPS-D7). The results showed that the sucrose preference, as a way to observe anhedonia, was not affected in both WT and *Fkbp5*-KO mice (Fig. 1C). The OFT and EPM tests were performed to examine anxiety-like behavior and locomotor activity. WT mice did not exhibit significant alterations in their anxiolytic responses, as indicated by the time spent in the central zone of the open field (Fig. 1D, E) and in the open arm in the EPM (Fig. 1G, H) in either the saline or LPS injection group. By contrast, LPS-injected *Fkbp5*-KO mice exhibited significant anxiety-like behavior, as evidenced by spending less time in the central zone (Fig. 1E), and no effect on the total distance moved was observed (Fig. 1F). In the EPM test, we found that *Fkbp5*-KO but not WT mice exhibited anxiety-like behavior with less time spent in the open arm after LPS injection (Fig. 1H). The total distance did not differ between the saline and LPS groups in both WT and *Fkbp*5-KO mice (Fig. 1I). Since *Fkbp5* is a stress-inducible GR target gene, the mRNA levels of *Fkbp5* were measured in LPS-injected mice. The result showed that the expression levels of *Fkbp5* mRNA were increased in both the hippocampus and liver on LPS-D1 and LPS-D7 (Fig. 1J, K). In addition, LPS increased the FKBP51 protein level in the hippocampus on LPS-D7 (Fig. 1L, Additional file 1: Fig. S3A).

Together, these results suggest that transient peripheral inflammation in mice can induce the *Fkbp5* gene expression but not anxiety or anhedonia after recovery from the sickness; whereas *Fkbp5* deficiency causes heightened anxiety response following peripheral inflammation.

### *Fkbp5* deletion reduced LPS-induced central immune responses in the hippocampus

**Fig. 2:**
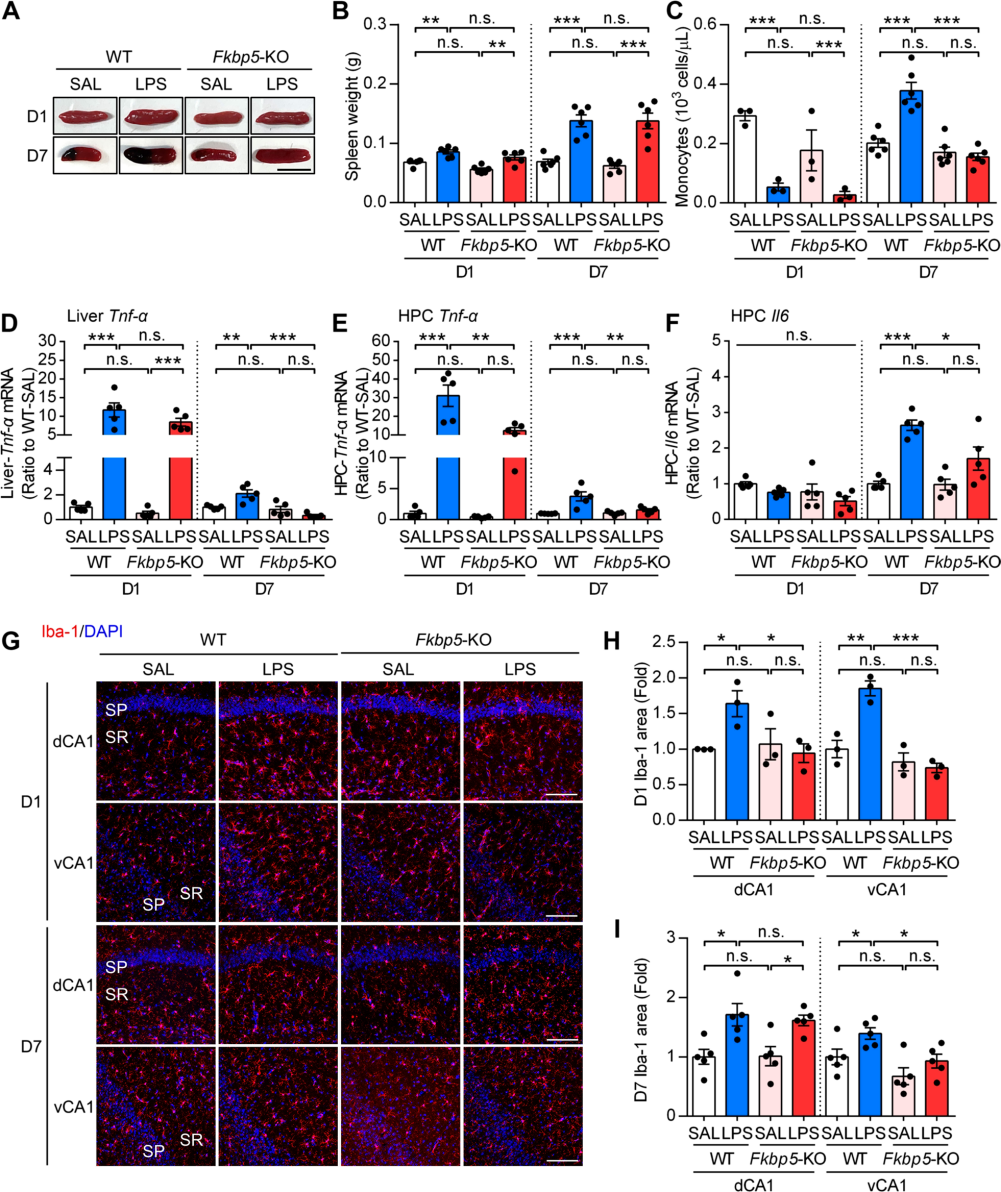
*Fkbp5* deletion attenuates LPS-induced hippocampal immune responses. **A**, **B** Spleens were dissected and weighed on day 1 and 7 post-injection of SAL or LPS (n = 6 mice per group). Scale bar: 1 cm. **C** Complete blood count analysis of peripheral blood samples collected from mice 1 or 7 days after saline or LPS injection. The monocyte counts are shown (D1, *n* = 3 mice per group; D7, *n* = 6 mice per group). **D–F** The liver and HPC of WT and *Fkbp5*-KO mice were analyzed by qRT-PCR for liver *Tnf-α* (**D**), HPC *Tnf-α* (**E**), and HPC *Il-6* (**F**) mRNA. Note that *Tnf-α* expression was induced on day 1 in both the liver and HPC, whereas delayed induction of *Il-6* in the HPC was observed on day 7. Data are indicated as the mean ± SEM. *p < 0.05, **p < 0.01, and ***p < 0.001 for the comparison between two groups in a two-way ANOVA followed by the Tukey post hoc test. **G** Representative immunofluorescence images of microglial marker Iba-1 (red) and DAPI (blue) in the mouse hippocampal dorsal and ventral CA1 (dCA1 and vCA1). Scale bar: 100 μm. **H**, **I** Quantification of the area expressing Iba-1 in the stratum pyramidale (SP) and stratum radiatum (SR) of the hippocampal CA1 region (D1, *n* = 3 mice per group; D7, *n* = 5 mice per group). Data are indicated as the mean ± SEM. *p < 0.05, **p < 0.01, and ***p < 0.001 for the comparison between two groups in an unpaired Student’s *t* test

To determine the effect of *Fkbp5* deletion on peripheral and central immune responses that could be involved in the mechanisms of anxiety responses following peripheral inflammation. We assessed the peripheral inflammatory process by analyzing the spleen weights and complete blood counts (CBC) analysis of peripheral blood samples collected from SAL or LPS-injected mice, and found that both LPS-D1 and LPS-D7 increased the spleen weights in both WT mice and *Fkbp5*-KO mice, especially in LPS-D7 (Fig. 2A, B). The CBC analysis revealed that the initial peripheral immune response to the LPS injection, i.e., LPS-D1, showed leukocytopenia and monocytopenia, and these responses were no difference between WT mice and *Fkbp5*-KO mice. (Fig. 2C, Additional file 1: Fig. S1). Notably, the number of monocytes was elevated in WT mice 7 days after the LPS injection, but this response was not observed in *Fkbp5*-KO mice (Fig. 2C). These results suggest that the LPS injection initially induced a suppressive effect to the peripheral immune cells that was not affected by the *Fkbp5* deletion. The elevation of immune cell counts was observed on LPS-D7, in which the peripheral monocyte elevation response was diminished by *Fkbp5* deficiency. However, we did not observe detectable leukocyte infiltration in the LPS-D7 hippocampus as assessed by the immunostaining of leukocyte marker CD45, with a positive control staining of the hypoxia ischemia brain injury indicating profound CD45^+^ infiltrating leukocytes in the hippocampus (Additional file 1: Fig. S2). We also found that LPS challenge used here did not induce detectable BBB leakage as assessed by the Evans Blue staining (Additional file 1: Fig. S2). Proinflammatory cytokine expression in the liver versus the hippocampus, which, respectively, represent the peripheral and central inflammatory responses, indicated that liver *Tnf*-*α* mRNA expression was increased in both WT and *Fkbp5*-KO mice on LPS-D1 and decreased on LPS-D7. Hepatic *Tnf*-*α* expression was significantly lower in *Fkbp5*-KO mice than in WT mice on LPS-D7 but not on LPS-D1 (Fig. 2D). Moreover, the *Tnf*-*α* mRNA expression in the hippocampus was increased on LPS-D1 and decreased on LPS-D7, and a considerably lower *Tnf*-*α* expression was observed in *Fkbp5*-KO mice than in WT mice at both time points (Fig. 2E). By contrast, a delayed increase in *Il-6* mRNA expression was observed on LPS-D7, and this effect was weaker in *Fkbp5*-KO mice than in WT mice (Fig. 2F). Ionized calcium-binding adapter molecule 1 (Iba-1) is the most common marker used for detecting the activated microglia and also infiltrating macrophages in the brain. The activation of microglia during an inflammatory response results in retraction of microglia processes and swelling of the microglia cell bodies. The morphological changes of microglia can be observed by the immunofluorescent signals of Iba-1. Importantly, immunofluorescence staining of Iba-1 showed that peripheral LPS injection induced microglial activation in both dorsal and ventral CA1 region of hippocampus on LPS-D1, and this effect was not observed in *Fkbp5*-KO mice (Fig. 2G, H). Similar to LPS-D1, the Iba-1-immunoreactive area was higher in WT mice than in *Fkbp5*-KO mice in ventral CA1 hippocampus on LPS-D7 (Fig. 2G, I). These LPS challenge-increased Iba^+^ cells mainly indicate activated microglia as the BBB leakage and leukocyte infiltration were not observed (Additional file 1: Fig. S2). Therefore, these results suggest that the peripheral LPS challenge used in this study induced microglial activation and elevation of proinflammatory response in the brain without inducing BBB leakage or peripheral leukocyte infiltration to the brain. *Fkbp5* deficiency significantly reduced the LPS-induced immune responses in the peripheral system as well as central nervous system.

### *Fkbp5* deletion reduced LPS-induced GR activation in the hippocampus

**Fig. 3:**
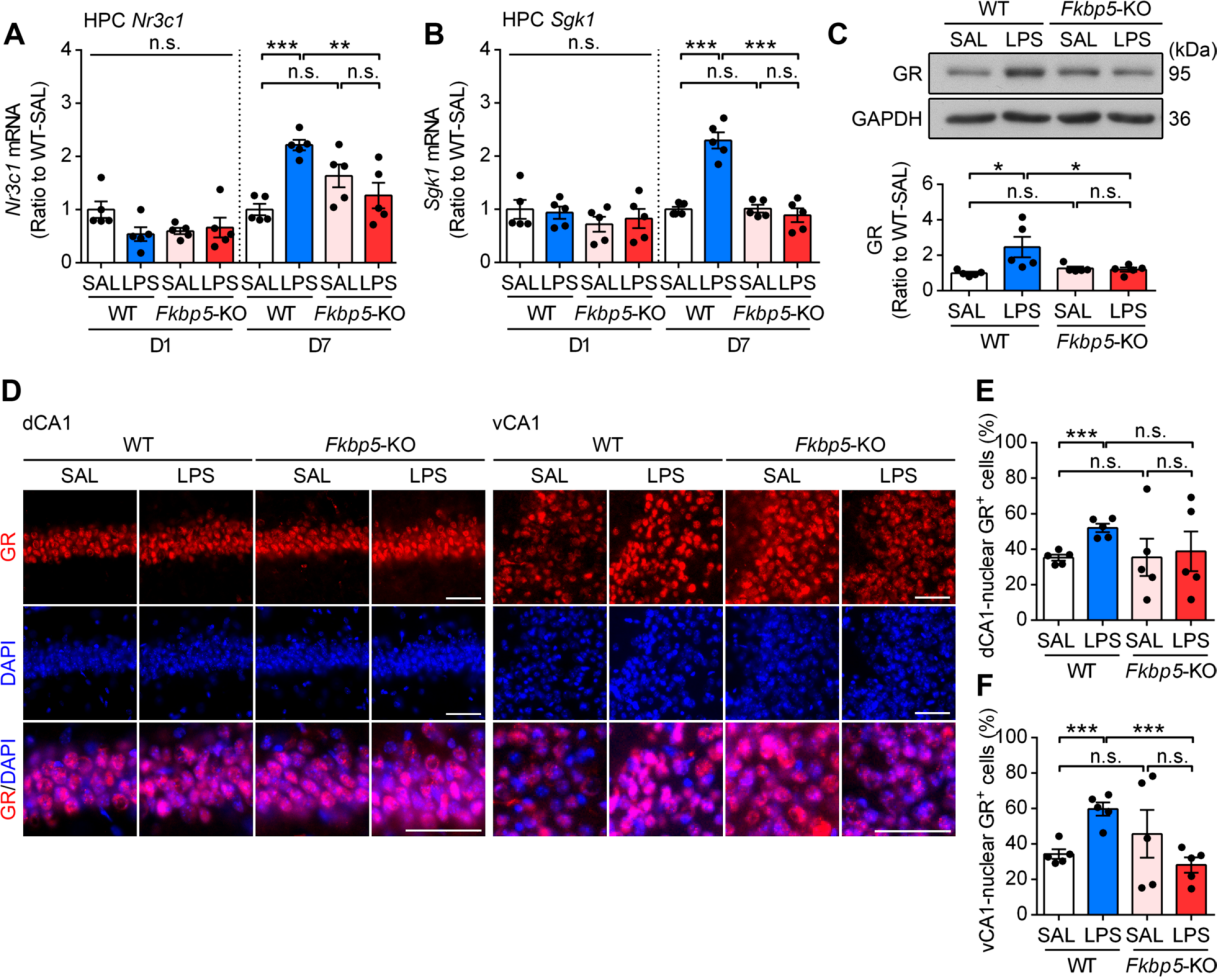
*Fkbp5* deletion attenuates LPS-induced GR activation. The HPC of WT and *Fkbp5*-KO mice were collected at days 1 and 7 after LPS injection, followed by mRNA and protein expression analysis. **A**, **B** qRT-PCR analysis of HPC *Nr3c1* (encoding GR) and *Sgk1* expression indicated that *Fkbp5* deficiency reduced the LPS-induced delayed upregulation of *Nr3c1* and *Sgk1* expression at day 7 in the HPC. **C** Western blotting indicated an increase in GR on day 7 after LPS injection in the HPC of WT but not *Fkbp5*-KO mice. The relative band intensities were normalized to GAPDH levels. Data are indicated as the mean ± SEM. *p < 0.05, **p < 0.01, and ***p < 0.001 for the comparison between two groups in a two-way ANOVA followed by the Tukey post hoc test. **D** Immunostaining of the GR in the mouse hippocampal dorsal and ventral CA1 (dCA1 and vCA1) along with counterstaining of the cell nucleus with DAPI. GR-positive cells were predominantly located in CA1 pyramidal cell layers. **E**, **F** The nuclear localization of the GR, which indicated the activated state of the GR, was quantified by overlapping GR-DAPI signals in the total DAPI count. Note that LPS significantly increased the number of nuclear GR^+^ cells in both the dCA1 and vCA1 of WT but not *Fkbp5*-KO mice. ***p < 0.001 for the comparison between two groups in an unpaired Student’s *t* test. *n* = 5 mice per group. Scale bar: 50 μm

Since GC/GR actions exert negative feedback effects on HPA activation in the hippocampus for stress adaptation [46]. We examined the expression and activation of hippocampal GR in mice after LPS injection. The result indicated that in parallel to the increase in *Il-6* mRNA expression, LPS injection induced a delayed increase in the expression of *Nr3c1* (encoding GR) and its transcriptional target gene *Sgk1* (serum/GC regulated kinase 1) on LPS-D7 but not LPS-D1 in the hippocampus of WT mice; both effects were diminished in *Fkbp5*-KO mice (Fig. 3A, B). In addition, LPS increased the GR protein level on LPS-D7 in the hippocampus of WT but not *Fkbp5-*KO mice (Fig. 3C, Additional file 1: Fig. S3B). Furthermore, we performed GR immunostaining in the mouse hippocampus, in which dorsal and ventral CA1 neurons receive the HPA axis–derived GC feedback, to examine the nuclear localization of the GR after LPS injection. Immunofluorescent images indicated that the GR was expressed in the perinuclear region in most CA1 pyramidal neurons in saline-injected WT mice and that LPS injection significantly increased nuclear GR-expressing neurons in both dorsal and ventral CA1 in WT mice (Fig. 3D–F). In the hippocampus of *Fkbp5*-KO mice, nuclear GR-expressing CA1 neurons appeared to considerably vary in both dorsal and ventral subregions, implying that the lack of FKBP51 might increase the individual variation in HPA axis activation. Moreover, *Fkbp5*-KO mice exhibited a considerably weak response to nuclear GR-expressing neurons, which became more numerous, after LPS induction in both dorsal and ventral CA1 (Fig. 3E, F).

These results jointly indicate that *Fkbp5* deficiency reduced peripheral inflammation-induced central immune responses as well as GR activation and upregulation in hippocampal regions exhibiting HPA axis feedback.

### PET/MR neuroimaging indicated that the susceptibility of limbic regions to LPS-induced neuroinflammation is FKBP51-dependent

**Fig. 4:**
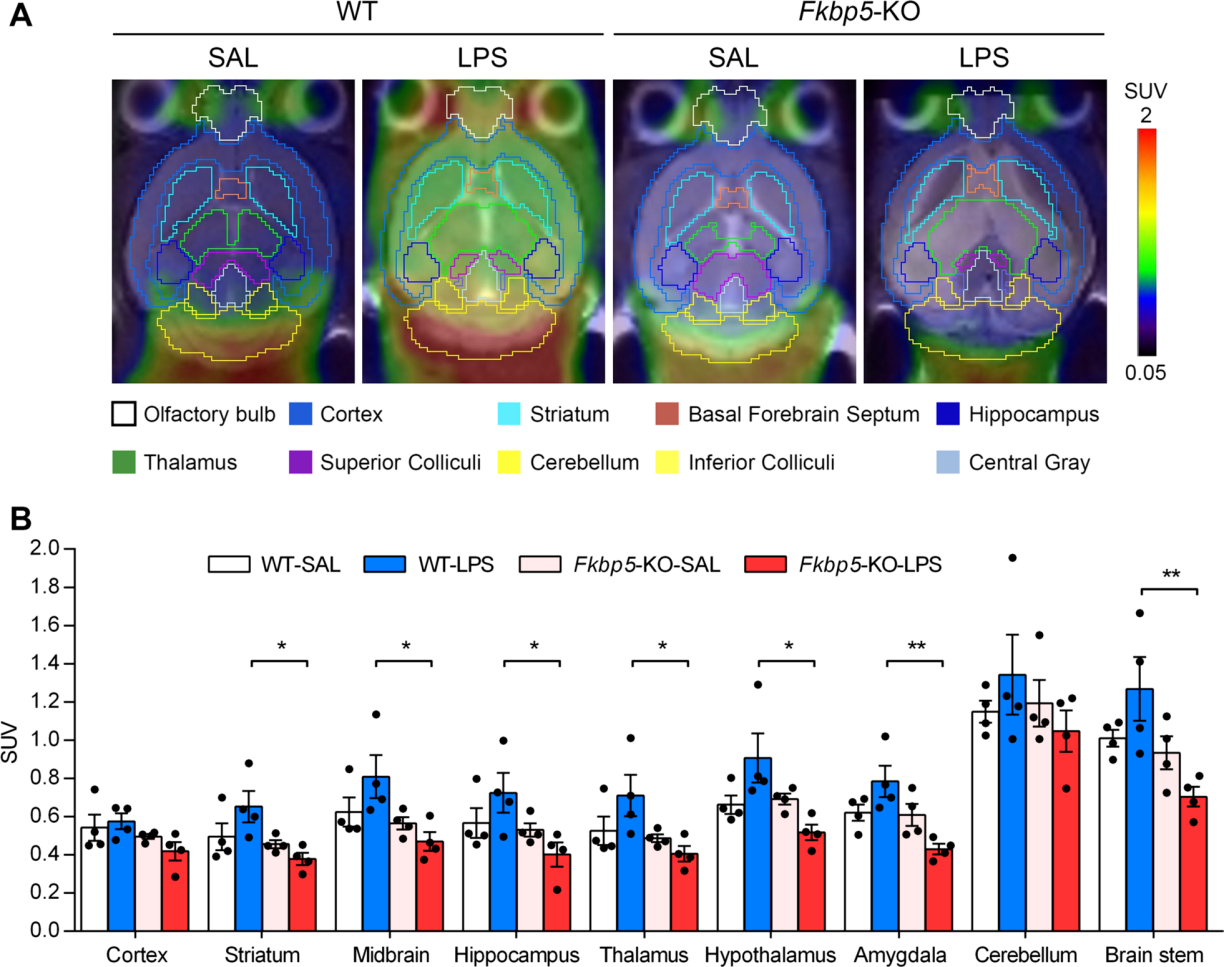
Neuroimaging of LPS-induced neuroinflammation in WT and *Fkbp5*-KO mice. **A** Representative small-animal 7-T PET/MRI of brain inflammation and TSPO radioligand [^18^F]-FEPPA imaging in WT versus *Fkbp5*-KO mice 7 days after LPS injection indicated that *Fkbp5* deficiency reduced glial activation and neuroinflammation. **B** Quantitation of [^18^F]-FEPPA SUV (standard uptake value) in different brain regions. A marked decrease was observed in brain cells expressing TSPO in the mouse brain 7 days after the administration of LPS in *Fkbp5*-KO mice compared with WT mice. Data are expressed as the mean ± SEM (*n* = 4 mice per group). *p < 0.05, and **p < 0.01 for the comparison between two groups based on two-way ANOVA followed by the Tukey post hoc test

To overcome the limitations of biochemical and histological examination in identifying *Fkbp5*-KO-affected brain regions in mice after inflammation, we performed in vivo 7-T whole-brain PET/MR neuroimaging by using [^18^F]-FEPPA, a radioligand of 18-kDa TSPO expressed on microglia, to measure the neuroinflammation in mouse brains collected on LPS-D7 [43, 47, 48]. An increase in the expression of Iba-1 and TSPO both used as biomarkers for microglial activation. The representative images of each group indicated that the cerebral distribution of [^18^F]-FEPPA was lower in *Fkbp5*-KO mice in the whole brain and hippocampus (Fig. 4A). Similar results were observed regarding the quantitative [^18^F]-FEPPA standard uptake value (SUV), indicating that LPS increased TSPO expression in the brains of WT mice universally, and the effect was occluded by *Fkbp5* deficiency, preferentially in limbic structures such as the amygdala, hypothalamus, and hippocampus (Fig. 4B). These results were consistent with the immunofluorescence staining of Iba-1 (Fig. 2G). Thus, the hippocampus and additional limbic regions, such as the amygdala, are affected by FKBP51-dependent neuroinflammation after peripheral inflammation.

### LPS induced hippocampal GAD65 upregulation in WT but not *Fkbp5*-KO mice

**Fig. 5:**
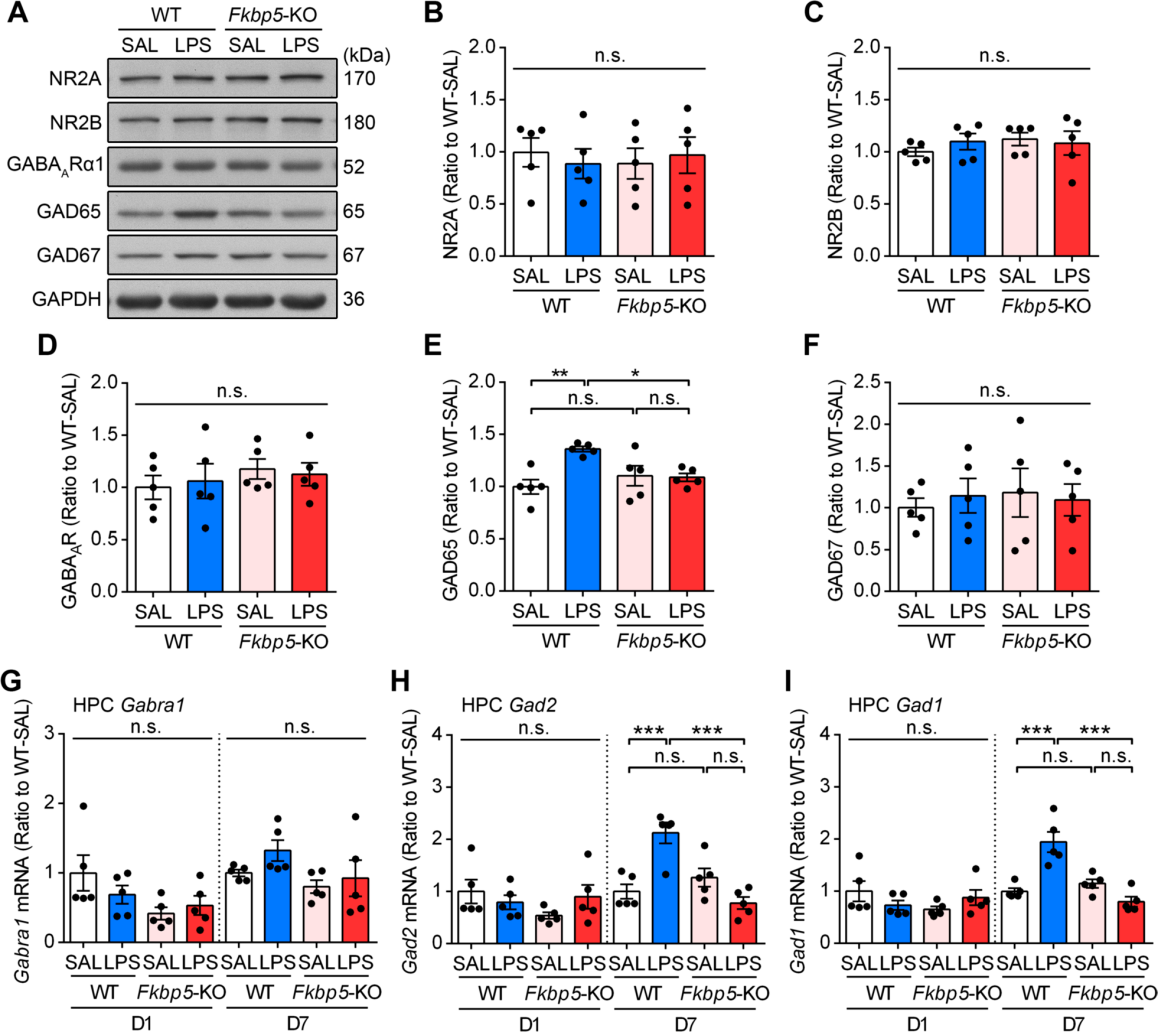
LPS increases GAD65 expression in the hippocampus of WT but not *Fkbp5*-KO mice. The hippocampal tissues of WT and *Fkbp5*-KO mice were collected 7 days after the injection of SAL or LPS. **A–F** Western blotting and quantification were performed to examine the expression of NMDA receptor subunits (NR2A, **B**; and NR2B, **C**), GABA_A_Rα1 (**D**), GAD65 (**E**), and GAD67 (**F**). The quantification of band densities was indicated by normalizing it to the GAPDH level. qRT-PCR analysis of *Gabra1* (**G**), *Gad1* (**H**), and *Gad2* (**I**) expression. LPS increased both the protein and gene expression of GAD65 in the HPC of WT mice, and these effects were reversed by the knockout of *Fkbp5*. Data are expressed as the mean ± SEM (*n* = 5 mice per group). *p < 0.05, **p < 0.01, and ***p < 0.001 for the comparison between two groups based on two-way ANOVA followed by the Tukey post hoc test

Anxiety disorders have been linked to neurotransmission imbalance. Given that dysregulated excitatory and inhibitory neurotransmission plays a role in anxiety behaviors [49], we examined the expressions of the excitatory *N*-methyl-d-aspartate (NMDA) receptor and inhibitory GABA_A_ receptor in the hippocampus of mice on LPS-D7. The Western blotting results indicated that the NMDA receptor NR2A and NR2B subunits and the GABA_A_ receptor α1 subunit (GABA_A_R_α1_) present in the hippocampus were not significantly affected after LPS injection in both WT and *Fkbp5*-KO mice (Fig. 5A–D, Additional file 1: Fig. S3C). GAD65 and GAD67 are GABA-synthesizing enzymes, and we found that the level of GAD65 but not GAD67 was significantly higher in the hippocampus of WT but not *Fkbp5-*KO mice after LPS induction (Fig. 5A, E, F, Additional file 1: Fig. S3C). The qRT-PCR analysis of the hippocampal gene transcription of *Gabra1* (encoding GABA_A_R_α1_), *Gad2* (encoding GAD65), and *Gad1* (encoding GAD67) on LPS-D1 and LPS-D7 indicated that LPS treatment did not affect *Gabra1* mRNA expression (Fig. 5G); however, both *Gad2* and *Gad1* mRNA expression levels were notably increased on day 7 in LPS-injected WT mice (Fig. 5H, I). Similar to its effect on IL-6 and GR/SGK1 induction, *Fkbp5*-KO abolished LPS-increased hippocampal *Gad2* transcription (Fig. 5H). These findings indicated that *Fkbp5* deficiency abolished LPS-induced GAD65 upregulation in the hippocampus, suggesting that FKBP51 mediates the inflammation-induced enhancement of GABAergic neurotransmission.

### Chronic GC intake induced FKBP51-dependent anxiety without upregulating hippocampal GAD65

**Fig. 6:**
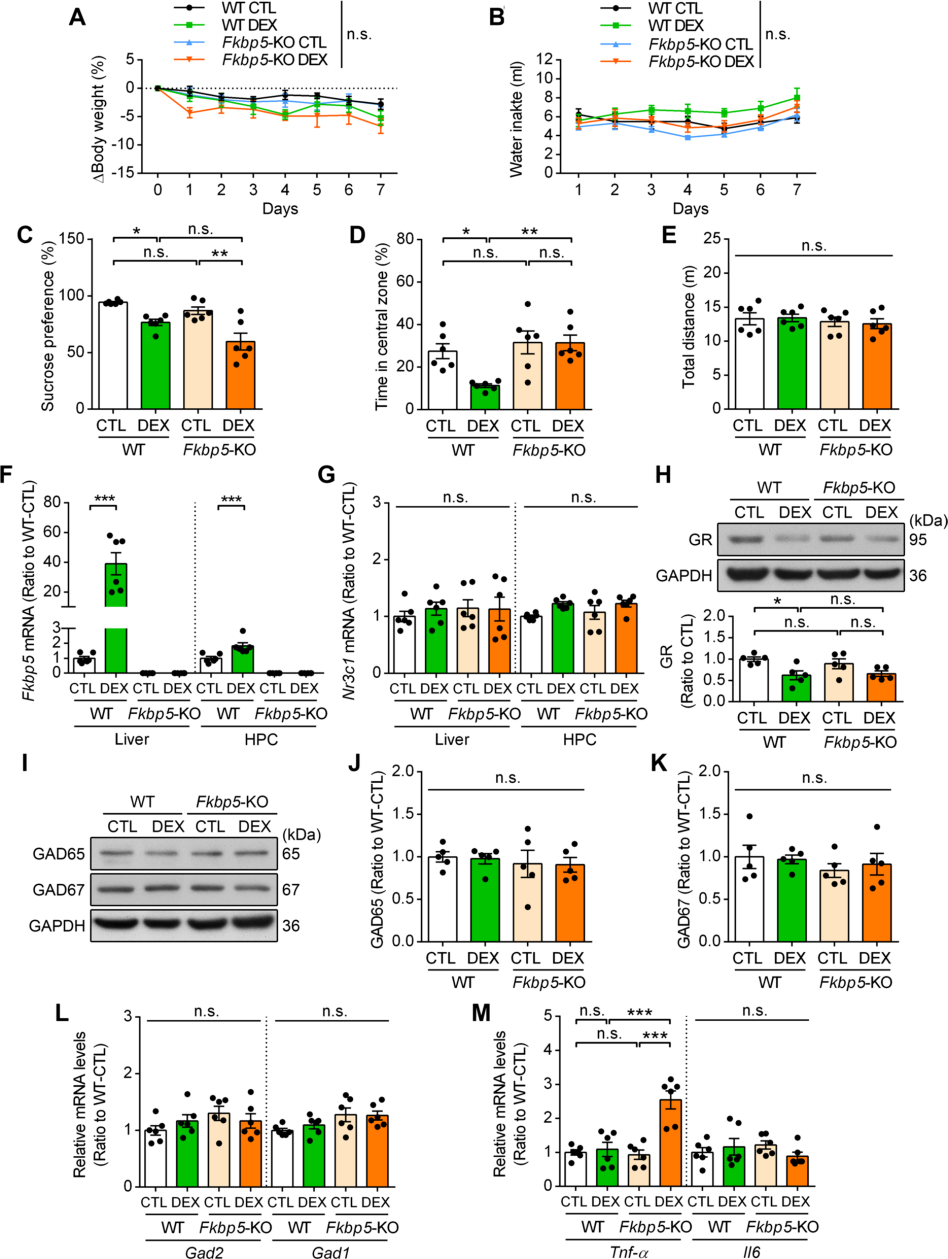
Chronic GC intake induces FKBP51-dependent anxiety without upregulating hippocampal GAD65. Behavioral assessment and tissue collection were performed in mice with free drinking access to tap water (CTL) or dexamethasone (DEX, 0.016 mg/mL) for 7 days. **A**, **B** The body weight and water intake of mice during DEX challenge were tracked for 7 days (n = 6 mice per group). **C–E** Mice were assessed using the sucrose preference test (**C**) and open-field test (**D**, **E**) on day 7 after DEX intake (*n* = 6 mice per group). **F**, **G** qRT-PCR analysis of liver and hippocampal *Fkbp5* and *Nr3c1* mRNA levels (*n* = 6 mice per group). **H–K** Western blot analysis of GR (**H**), GAD65 (**J**), and GAD67 (**K**) expressions in the HPC (*n* = 5 mice per group). The band intensity was quantified after it was normalized to the GAPDH level. **L, M** qRT-PCR analysis of hippocampal *Gad2*, *Gad1*, *Tnf-α* and *Il-6* mRNA levels (*n* = 6 mice per group). Data are expressed as the mean ± SEM. *p < 0.05, **p < 0.01, and ***p < 0.001 for the comparison between two groups in a two-way ANOVA followed by the Tukey post hoc test

The overall effect of *Fkbp5* deletion on LPS-induced neuroinflammation and stress response suggested that FKBP51 is involved in the early and delayed phases of proinflammatory responses and even stress resilience after peripheral inflammation. Because LPS-induced GAD65 upregulation may contribute to *Fkbp5*-dependent stress resilience, we examined whether direct GC elevation-induced noninflammatory stress would increase hippocampal GAD65 expression. A free-drinking DEX model was used in this study to increase systemic GC and activate the HPA axis without inducing physical stress. During 7-day DEX drinking, both WT and *Fkbp5*-KO mice showed trend of decreasing body weight and increasing water intake as compared to their respective tap water drinking control, and no significant difference was observed among the four groups by two-way ANOVA analysis (Fig. 6A, B); this finding is in contrast to that of LPS-induced sickness (Fig. 1). Therefore, the DEX-drinking model did slight induce glucocorticoid-induced metabolic effect [50], and this effect was not affected by *Fkbp5* deletion. We performed the sucrose preference test and OFT to assess anhedonia and anxiety-like behavior, respectively. We found that DEX decreased the sucrose preference and the time spent in central zone in WT mice group (Fig. 6C, D), supporting the previous notion that glucocorticoid-mediated stress response induces depressive-like and anxiety-like behavior, respectively. *Fkbp5*-KO did diminish the DEX-induced anxiety-like (Fig. 6D), but not depressive-like (Fig. 6C), behavior. Furthermore, no difference was noted in the distance traveled in the OFT among the four groups (Fig. 6E). This result is in accordance with the previous notion that *Fkbp5* mediates stress-induced anxiety. We also verified the *Fkbp5* mRNA expression in this model that was substantially increased in the liver (81.6-fold) and moderately increased in the hippocampus (1.8-fold; Fig. 6F). These results indicated that *Fkbp5* deficiency diminished anxiety-like behavior in the GC-induced stress model, which differs from the inflammation-induced anxiogenic effect observed in *Fkbp5*-KO mice (Fig. 1), suggesting the anxiety following inflammation (particularly innate immunity) is distinctly regulated by a set of neuro-mechanisms from that following the GC-induced stress.

In the hippocampus, chronic DEX intake did not affect hippocampal GR mRNA or protein levels and even reduced the GR protein level (Fig. 6G, H, Additional file 1: Fig. S4A) possibly due to activation-induced proteasome degradation [51, 52]; this finding differs from that on the increase in the GR level observed in the LPS model that was not affected by *Fkbp5* deficiency. Similarly, chronic DEX intake did not affect the mRNA and protein expression of GAD65 and GAD67 in the hippocampus of WT or *Fkbp5*-KO mice (Fig. 6I–L, Additional file 1: Fig. S4B). In addition, chronic DEX did not induce proinflammatory *Tnf*-*α* and *Il-6* expression in the hippocampus, and *Fkbp5* deficiency even increased *Tnf*-*α* expression in DEX-treated mice (Fig. 6M).

These results jointly indicate that, unlike peripheral inflammation, the GC-induced HPA axis activation that induced anxiety and depressive behaviors is not accompanied with hippocampal neuroinflammation or GAD65 upregulation. *Fkbp5* deficiency exerted an anxiolytic effect on chronic GC-induced anxiety and increased proinflammatory gene expression but had no effect on GABAergic gene expression, which is opposite to that of its effect on the LPS model.

### *Fkbp5* deficiency reduced peripheral inflammation-induced GAD65 alterations in the hippocampus and amygdala

**Fig. 7:**
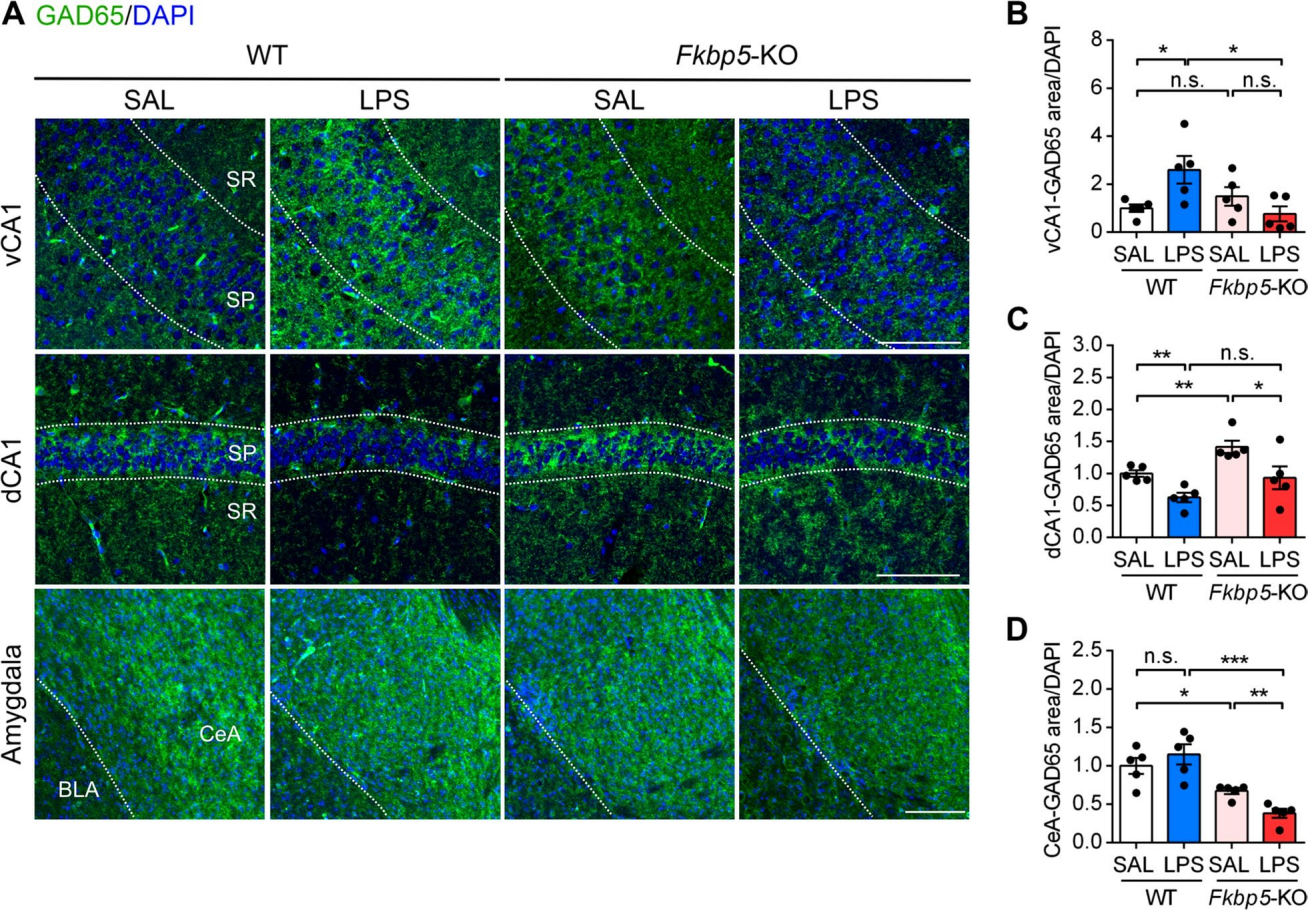
Subregional alterations in GAD65 expression in the hippocampus and amygdala in LPS-injected mice. Mouse brains were harvested 7 days after the injection of SAL or LPS for immunohistochemical analysis. **A** Representative immunofluorescent image of GAD65 (green) and DAPI (blue) staining in the hippocampal subregion of the ventral CA1 (vCA1, upper panel) and dorsal CA1 (dCA1, middle panel) as well as the amygdala (lower panel). **B–D** The area expressing GAD65 was quantified by calculating the ratio of their immunoreactive area to total number of DAPI in the stratum pyramidal (SP) layer of vCA1 (**B**), dCA1 (**C**), or central nucleus of the amygdala (CeA, **D**). Data are indicated as the mean ± SEM (*n *= 5 mice per group). *p < 0.05, **p < 0.01, and ***p < 0.001 for the comparison between two groups in an unpaired Student’s *t* test. Scale bar: 100 μm. SR: stratum radiatum, BLA: basolateral amygdala

To better understand whether LPS-induced GAD65 upregulation occurred in anxiety-related brain regions [53], we performed immunofluorescence staining analysis to examine GAD65 expression in the ventral and dorsal hippocampus and amygdala of LPS-D7 mice. In immunofluorescent confocal images, punctate GAD65 immunoreactivity appeared to indicate GABAergic presynaptic terminals, and we found that LPS-injected WT mice exhibited opposite changes of GAD65 immunoreactivities in the ventral versus dorsal CA1; the signals were stronger in the ventral CA1 but considerably weaker in the dorsal CA1 compared with their signals in saline-injected WT mice (Fig. 7A, B). Notably, the LPS-induced increase in GAD65 in the ventral CA1 was reversed in *Fkbp5*-KO mice that exhibited lower GAD65 expression than did saline-injected *Fkbp5*-KO mice (Fig. 7B). Furthermore, the LPS-induced decrease in GAD65 in the dorsal CA1 was diminished in *Fkbp5*-KO mice that exhibited higher basal level of GAD65 than WT mice (Fig. 7C). In addition, we examined GAD65 expression in the amygdala and found that GAD65 was abundantly expressed in the central nucleus of the amygdala (CeA), but the levels were not altered in LPS-treated WT mice, whereas GAD65 expression was decreased further in LPS-treated *Fkbp5*-KO mice (Fig. 7A, D). These findings indicated that FKBP51 might function to maintain GAD65 level in the amygdala under inflammatory challenge. Furthermore, the results suggested that GAD65 expression, which reflects the capacity for GABA synthesis, was differentially altered by peripheral LPS injection-induced stress and neuroinflammation in the observed anxiety-associated brain regions, thus indicating that FKBP51 may mediate multiregional changes in GABA synthesis for coping with stress under inflammation.

### *Fkbp5* deficiency abrogated the inhibitory effect of LPS on neuronal activity in ventral CA1

**Fig. 8:**
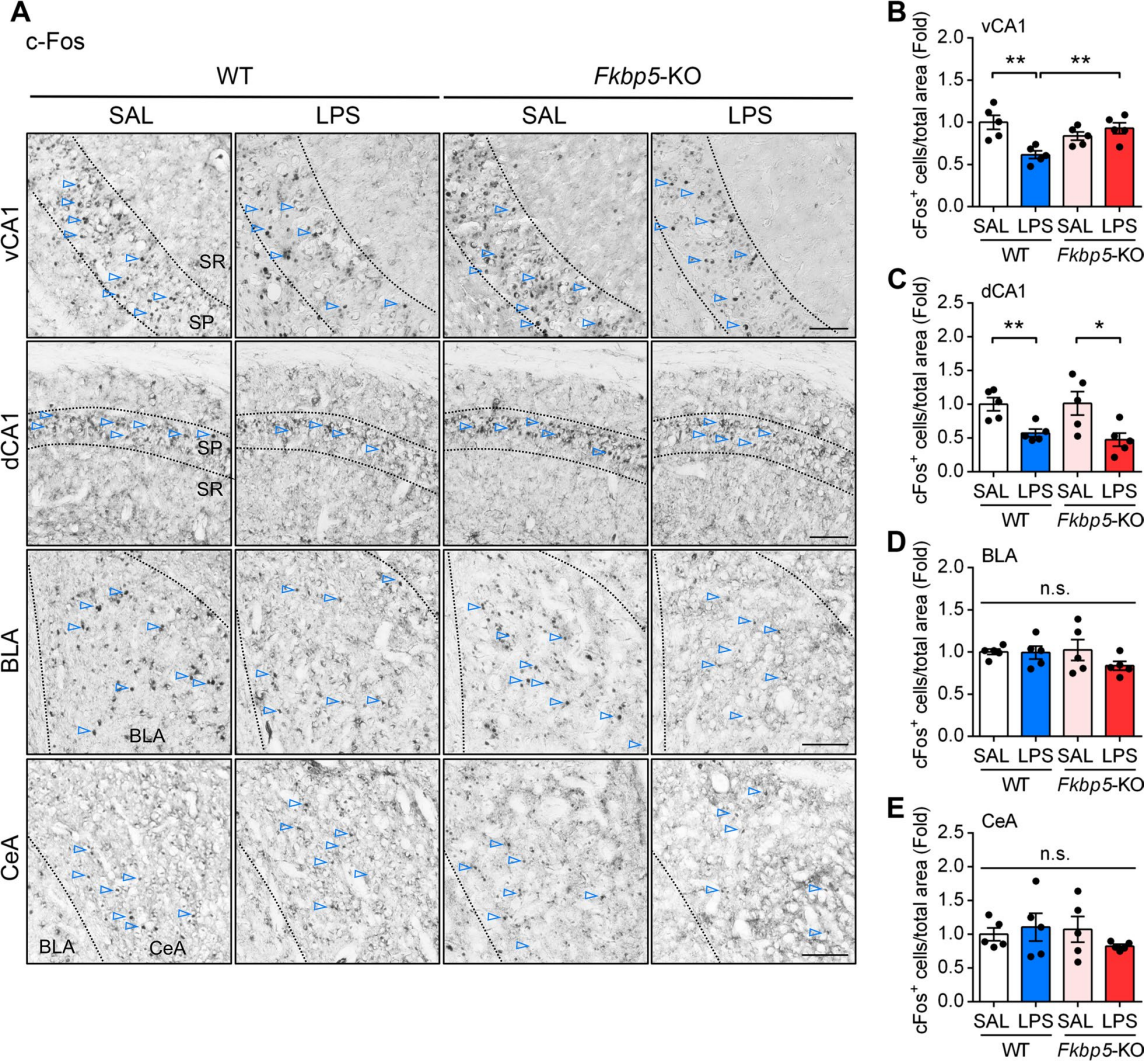
*Fkbp5* deficiency LPS-reduced c-Fos expression in the hippocampus in ventral CA1. WT and *Fkbp5*-KO mouse brains were collected 7 days after the intraperitoneal injection of SAL or LPS. **A** Representative immunofluorescent images of the neuronal activity marker c-Fos (arrowheads) in the hippocampal subregion of the ventral CA1 and dorsal CA1 (vCA1 and dCA1, upper panel) as well as the amygdala (BLA and CeA, lower panel). **B–E** The c-Fos-labeled neurons were quantified by calculating the percentage of c-Fos-positive cells in the region of interest in the total area of the vCA1 (**B**), dCA1 (**C**), BLA (**D**), and CeA (**E**). Data are indicated as the mean ± SEM (*n* = 5 mice per group). *p < 0.05, and **p < 0.01 for the comparison between two groups in an unpaired Student’s *t* test. Scale bar: 100 μm

We hypothesized that the alteration in GAD65 expression following LPS injection would alter neuronal activity. Therefore, we examined the expression of the nuclear protein c-Fos, an immediate early gene widely used as a marker for activated neurons [54], in the hippocampus and amygdala. We found that c-Fos decreased in both the ventral and dorsal CA1 subregions on LPS-7D, and only the effect on ventral CA1 was absent in *Fkbp5*-KO mice (Fig. 8A, B). We also examined the c-Fos expression in the basolateral amygdala (BLA) and CeA subregions that are involved in anxiety behavior, and found no effect of LPS on their neuronal activity (Fig. 8A, D, E). It is noted that LPS also suppressed high frequency stimulation-induced long-term potentiation (LTP) on dorsal CA1, and this effect can be blocked by *Fkbp5* deletion (Additional file 1: Fig. S5). No significant change in neuronal cell viability between SAL- and LPS-treated groups was observed in these regions of interest in WT and *Fkpb5*-KO hippocampus as indicated by immunostaining of neuronal marker NeuN (Additional file 1: Fig. S6). These findings suggested that peripheral inflammation suppressed neuronal activity in both ventral and dorsal CA1 even after recovery from sickness, and the response in ventral CA1 was FKBP51-dependent and correlate with the elevation of GABA-synthesizing GAD65. FKBP51 in dorsal CA1 may account for the inhibitory effect of peripheral inflammation on synaptic plasticity, not neuronal activity.

## Discussion

**Fig. 9:**
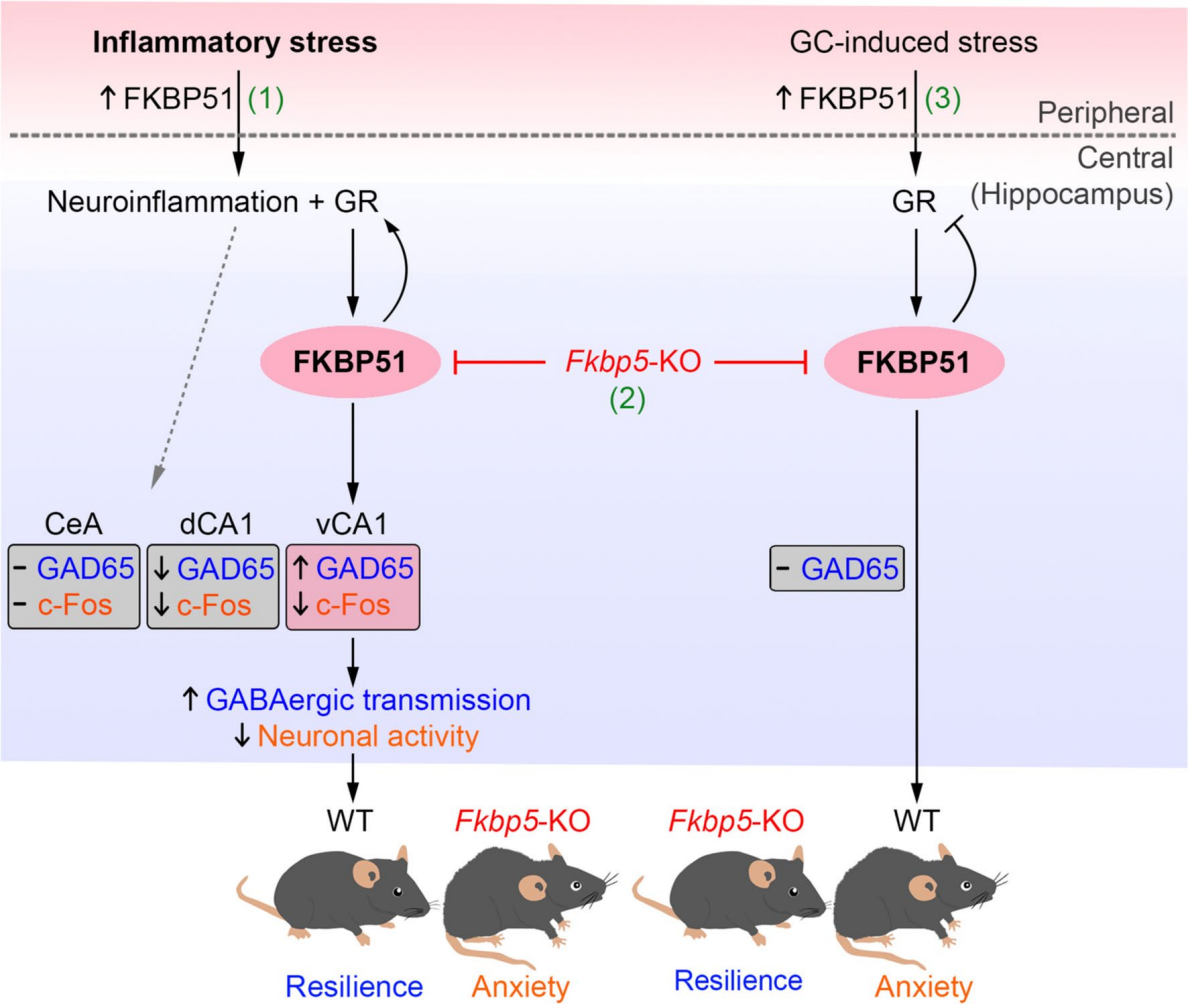
Schematic diagram showing that FKBP51 mediates inflammation-associated stress adaptation. The scheme illustrates the underlying mechanism of FKBP51-mediated stress adaptation after systemic inflammation. (1) In the inflammation-induced stress model, peripheral LPS stimulation causes activation of the GR signaling that upregulates FKBP51 as well as neuroinflammation, accompanied with upregulation of GAD65 for GABA synthesis, and suppression of c-Fos-indicated neuronal activity, in the ventral hippocampal CA1 subregion; whereas both GAD65 and neuronal activity were reduced in dorsal CA1. The mice under this condition appear to be resilient to inflammatory stress. (2) Although FKBP51 deficiency attenuates the LPS-induced GR expression and proinflammatory cytokines in the hippocampus, such deficiency results in the development of inflammation-induced anxiety, likely via blocking the GAD65 upregulation to de-suppress the neuronal activity in the ventral CA1. In contrast, FKBP51 deficiency does not block the LPS-induced suppression of GAD65 and neuronal activity in dorsal CA1. (3) GC-induced HPA axis hyperactivity does not increase GAD65 expression, thus resulting in the development of anxiety. *Fkbp5* deficiency exerted an anxiolytic effect by regulating GR sensitivity in GC-induced stress. Taken together, FKBP51 modulates stress-induced anxiety in various stressors, and plays a critical role in stress adaptation and GABAergic neurotransmission after inflammation in the ventral hippocampus. (↑: increased, ↓: decreased, -: unchanged)

In this study, we unveiled an essential role of FKBP51 in the inflammation-induced anxiety through GABAergic system in the brain, particularly in the hippocampus. Although increased FKBP51 expression and inflammatory responses have been, respectively, considered as potential risk factors for anxiety disorders [13, 55], our results extend beyond current knowledge by clarifying the role of FKBP51 in anxiety mechanisms following inflammation. We found that *Fkbp5* deletion produced a strong anxiety response after recovery from LPS-induced sickness. *Fkbp5* deletion reduced the expressions of TNF-α and IL-6 and the levels of neuroinflammatory markers in the hippocampus following LPS treatment. In addition, the upregulation and activation of GR and the subsequent upregulation of GAD65 in the ventral hippocampus observed in the hippocampus of WT mice following LPS treatment were not noted in *Fkbp5*-KO mice. Alterations in the expression of GR and GAD65 that were likely to be specific to the inflammation model but not the GC-induced stress model indicated that *Fkbp5* deletion abrogated LPS-enhanced GAD65 expression in the ventral CA1 of the hippocampus and impaired GR/HPA stress signaling. In contrast, *Fkbp5* deletion attenuated GC-induced anxiety without affecting hippocampal GAD65 expression. The deduced mechanism of FKBP51-mediated stress adaptation under inflammation versus stress conditions is illustrated in Fig. 9 and discussed as follows.

Accumulating evidence has suggested a link between inflammation and anxiety [23, 56]. LPS stimulation caused the innate immune system to trigger the release of proinflammatory cytokines both in the peripheral and CNS and potentially resulted in anxiety-like behavior [57, 58]. In healthy volunteers treated with an endotoxin, a significant correlation was found between cortisol secretion and anxiety [59]. These preclinical and clinical data jointly suggest that the HPA axis plays a role in the endotoxemia–anxiety relationship [60]. Because GR is a crucial determinant of the negative feedback of cortisol response to HPA axis function, the key regulator FKBP51 might participate in inflammation-related anxiety behavior. In our transient systemic inflammation model, we found that the anxiety level of *Fkbp5*-KO mice was higher than that of WT mice despite both these groups exhibiting similar transient sickness behavior. FKBP51 might mediate the optimal inflammatory responses for physiological adaptation against inflammation-induced anxiety. Furthermore, the findings from TSPO neuroimaging revealed that most of the brain regions exhibited FKBP51-dependent neuroinflammation. On the basis of this finding, future studies can investigate the region-specific effect of FKBP51 on inflammation-induced alterations in neurotransmission and subsequent adaptive behaviors.

Genetic association studies conducted in humans have reported an association of FKBP51 with susceptibility to stress-related anxiety disorders [3, 61]. In mice, the manipulation of FKBP51 modulated stress-related anxiety behavior. For example, FKBP51 antagonist treatment suppressed the stress-induced anxiogenic phenotype [7]. Similarly, a prior study demonstrated that the attenuated induction of *Fkbp5* expression reduced anxiety behavior [8, 62]. These findings accord with our observations for the GC-intake stress model that demonstrated an association of the loss of FKBP51 with an enhanced anxiolytic response following stress, suggesting that FKBP51 ablation disrupted the adaptive neuroendocrine response to stress, thus impairing coping behavior and causing anxiety. However, in the present study, *Fkbp5*-KO mice exhibited higher vulnerability to inflammation-associated anxiety. This finding suggested that FKBP51 mediates the dissimilar regulation of the anxiety response when different stressors are encountered, where the anxiety-promoting effect is likely to be specific to inflammation.

Compared with LPS-treated WT mice, LPS-treated *Fkbp5*-KO mice appeared to be more anxious and exhibited non-significant changes in GR expression in the hippocampus. These observations are compatible with that of a recent study indicating that after acute stress exposure, changes in GR expression were non-significant in *Fkbp5*-deficient mice, suggesting that *Fkbp5*-deficient animals are deprived of a static calibrator to regulate GR activity and subsequent endocrine stress reactivity [8]. However, the role of FKBP51 in anxiety behavior in the inflammation model has not been adequately investigated. Most studies on FKBP51 have used the GC-induced stress model. In response to stress, HPA axis activation-derived GC interacts with the GR in the hippocampus, thus resulting in the negative feedback of the HPA axis that maintains neuroendocrine homeostasis [63]. In our study, the regulation of hippocampal GR protein expression differed between LPS-injected and DEX-treated mice; LPS increased but DEX reduced GR expression in WT mice. Moreover, a recent study reported that both the protein expression and nuclear translocation of GR were increased in the hippocampus of resilient mice after exposure to social defect stress. By contrast, susceptible mice exhibited lower GR protein expression than did resilient mice [64]. These results are similar to our finding of WT mice exhibiting an increased expression and nuclear translocation of the GR and resilience to anxiety following LPS injection, whereas *Fkbp5*-KO mice exhibiting susceptibility to anxiety had a non-significant change in GR expression. These findings suggest that adequate GR protein expression was observed in response to inflammation in WT mice, resulting in the feedback inhibition of the HPA axis. The non-significant change in GR expression and the nuclear translocation of the GR in the hippocampus reflect the dysfunction of the HPA axis. The inflammation-induced upregulation and the nuclear translocation of the GR in the hippocampus might be correlated with resilience to anxiety for stress adaptation. The single LPS injection and chronic DEX intake model may, respectively, display distinct role of FKBP51 on anxiety. Nevertheless, given the complex mechanism of anxiety disorders, the comparison between these two stress models still provide new insight on how FKBP51 regulate anxiety when coping with different stress context.

As to the important finding on the TNF-α levels between *Fkpb5*-KO-CTL and *Fkpb5*-KO-DEX, it was reported that chronic HPA axis activation would induce neuroinflammation [65, 66]. Previous study indicated that stress stimulation or high level of GCs can promote the accumulation of inflammatory mediator in the brain. This stress-induced inflammation has been attributed to the mechanism. Our finding indicated that DEX-induced hippocampal TNF-α expression can also be diminished in *Fkbp5-*KO mice, suggestion that FKBP51 mediate the GCs-augmented neuroinflammation in the brain in a similar fashion as to the LPS-induced neuroinflammation. But then, DEX-induced neuroinflammation is not accompanied with the GAD65 elevation in the hippocampus, suggesting that the neuroinflammation might not be the direct cause of elevation in GABAergic neurotransmission in the hippocampus.

GABAergic neurotransmission is closely linked with anxiety behavior [67]. However, the mechanisms underlying the effect of FKBP51 on GABAergic neurotransmission remain unclear. A recent study reported that *Fkbp5-*KO mice exhibited higher levels of GABA neurotransmitter and GAD65 expression in the hippocampus, resulting in increased frequency of miniature inhibitory postsynaptic currents [68]. However, our *Fkbp5*-KO mice exhibit a higher basal level of GAD65 in dorsal CA1 subregion of hippocampus, implicating the FKBP51 has regional effect on GAD65 expression. Moreover, FKBP51 may play a critical role in the regulation of GABAergic neurotransmission. Another study reported that mice with a deficiency in Delta-like 2 (a member of the NOTCH ligand family) gene (*Dlk2*) exhibited downregulated *Fkbp5* expression and increased vulnerability to anxiety-like behavior [69]. Furthermore, the deletion of *Dlk2* altered GABA receptor gene expression and inhibited the anxiolytic effect of benzodiazepines. These findings indicate a link between FKBP51 and GABAergic transmission. Our finding of the suppression of the LPS-induced expression of the GABA-synthesizing enzyme in *Fkbp5-*KO mice indicated that FKBP51 might regulate emotional behavior by participating in the response of the GABAergic system to stress.

The hippocampus is not only critical for cognitive processes in the dorsal CA1 subregion, but also implicated in the pathogenesis of mood and anxiety disorders in the ventral CA1 subregion [70]. The ventral hippocampus–amygdala is a crucial circuit involved in the regulation of anxiety behavior. In our study, we found that after being subject to LPS treatment, WT mice exhibited increased GR expression in the hippocampus and higher GAD65 expression in the ventral CA1. These results suggest that ventral hippocampal GABAergic neurotransmission is enhanced to adapt to an acute inflammatory event and reduce anxiety. However, these adaptive responses were not observed when *Fkbp5* was knocked out in mice. A previous study reported that GABA receptors are involved in the susceptibility of mice to LPS endotoxemia, and the effects are abolished by the GABA receptor blockade [71]. An in vitro study demonstrated that the exposure of hippocampal neurons to LPS enhanced the synaptic GABAergic input [72]. Furthermore, rats with septic shock exhibited an increased expression of the GABA_A_ receptor to the hippocampus [73]. These observations underscore a relationship between GABA activity and endotoxic inflammatory responses in the hippocampus. In addition to the established role of the hippocampus in buffering the stress response through the inhibition of the HPA axis, a recent study identified a hippocampus–hypothalamic circuit that rapidly controls anxiety-related behavior [37]. Considering these findings, we speculate that an intricate interplay between the HPA axis and hippocampus modulates anxiety and that GR signaling and FKBP51 participate in moderating the GABAergic function in the hippocampus. The LPS-induced GR increase in the hippocampus might be caused after LPS injection in the presence of FKBP51. The response of FKBP51 deficiency might interrupt this LPS effect on hippocampal GR expression and thus affect the behavior. Whether the increase in GR expression contributes to the upregulation of GABA neurotransmission in inflammation-induced brain adaptation should be explored in future studies.

## Conclusions

A plethora of evidence suggests that stress and inflammation account for the pathogenesis of mental disorders. In fact, many stress-related metabolic diseases, such as hypertension, obesity, and diabetes, have been associated with systemic inflammatory responses and share features of anxiety. Clinical evidence indicates that anxiety disorder is rather common in patients who exhibit a robust innate immune response following COVID-19 infection [74], further supporting a relationship between inflammation and anxiety. Although the role of FKBP51 in post-COVID-19 anxiety remains unclear, it acts as a regulator in the core biological pathway of the stress system (i.e., HPA axis) and subsequent emotional regulation. We speculate that compromised function of FKBP51 might contribute to inflammation-induced anxiety behavior, which deserves to be further investigated in distinct inflammation-associated diseases anxiety. In summary, our study results suggest that FKBP51 mediates GR signaling and GABAergic neurotransmission against both peripheral inflammation and pertinent anxiety behavior in ventral hippocampus. This information may help in identifying the potential neurobiological mechanisms underlying inflammation-related anxiety behavior and in developing therapeutic interventions.

## Supplementary Information

**Additional file 1.** Additional table and supplementary figures.

## Data Availability

The data that support the findings in this study are available from the corresponding author upon reasonable request.

## Abbreviations

ANOVA: Two-way analysis of variance
BLA: Basolateral amygdala
CeA: Central nucleus of the amygdala
CNS: Central nervous system
dCA1: Dorsal CA1
DEX: Dexamethasone
EPM: Elevated plus maze
FKBP51: FK506-binding protein 51
*Fkbp5*-KO: *Fkbp5*-Knockout
GABA: γ-Amino butyric acid
GABA_A_R_α__1_: GABA_A_ receptor α1 subunit
GAD65: Glutamic acid decarboxylase 65
GAD67: Glutamic acid decarboxylase 67
GC: Glucocorticoid
GR: Glucocorticoid receptor
HPA: Hypothalamic–pituitary–adrenal
HPC: Hippocampus
Iba-1: Ionized calcium-binding adapter molecule 1
IL-6: Interleukin-6
LPS: Lipopolysaccharide
LTP: Long-term potentiation
OFT: Open-field test
PET: Positron emission tomography
qRT-PCR: Quantitative real-time polymerase chain reaction
SAL: Saline
SGK1: Serum/GC regulated kinase 1
TNF-α: Tumor necrosis factor-α
TSPO: Translocator protein
vCA1: Ventral CA1
WT: Wild type
