# Supplementary material for: FKBP51 mediates resilience to inflammation-induced anxiety through regulation of glutamic acid decarboxylase 65 expression in mouse hippocampus
Source: J Neuroinflammation. 2022 Jun 15;19:152. doi: 10.1186/s12974-022-02517-8 (PMC9198626; doi:10.1186/s12974-022-02517-8)
Supplement: Supplementary file 1 — Additional file 1. Additional table and supplementary figures. [file 12974_2022_2517_MOESM1_ESM.docx]

**Additional file 1**

**Table S1. qRT-PCR primer list**

| Gene | Primer sequence (5’→3’) | NCBI accession No. |
| --- | --- | --- |
| *Fkbp5* | F: AAAGGACAATGACTACTGATGAGG | NM_010220.4 |
|  | R: CTGACAACATCCCTTTGTAGTGGAC |  |
| *Tnf-α* | F: TAGCCCACGTCGTAGCAAAC | NM_013693.3 |
|  | R: GCAGCCTTGTCCCTTGAAGA |  |
| *Il6* | F: GCCAGAGTCCTTCAGAGAGA | NM_031168.2 |
|  | R: TTAGCCACTCCTTCTGTGAC |  |
| *Nr3c1* | F: AGAATGACTCTACCCTGCATGTATGAC | NM_008173.4 |
|  | R: CTGGAGTTTCCTTCCCTTTTGACAATGGC |  |
| *Gabra1* | F: CACCATGAGGTTGACCGTGA | NM_010250.5 |
|  | R: CTACAACCACTGAACGGGCT |  |
| *Gad2* | F: TCCTTTCCTGGTGAGTGCCA | NM_008078.2 |
|  | R: AGTTGGCCCTCTCTACTCCAC |  |
| *Gad1* | F: ATCGCTCCACCAAGGTTCTG | NM_008077.5 |
|  | R: AAATCGAGGGTGACCTGTGC |  |
| *18s* | F: GAGGTGAAATTTCTTGGACCGG | NR_003278.3 |
|  | R: CGAACCTCCGACTTTCGTTCT |  |

F, Forward primer; R, Reverse primer


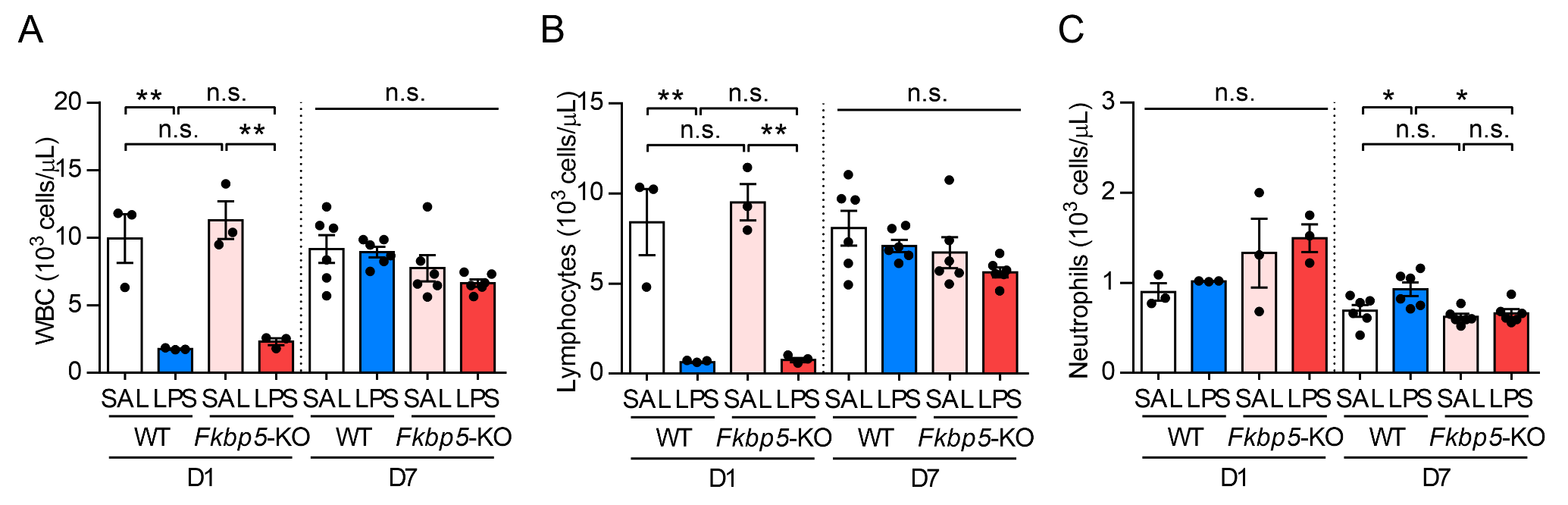


**Fig. S1 White blood cells parameters of WT and *Fkbp5*-KO mice on day 1 and 7 post-LPS injection.**

Complete blood count analysis of peripheral blood samples collected from mice 1 or 7 days after saline or LPS injection. White blood cell (WBC), lymphocyte, and neutrophil counts are shown (D1, n = 3 mice per group; D7, n = 6 mice per group). Data are expressed as the mean ± SEM. **p* < 0.05, ***p* < 0.01 for the comparison between two groups based on two-way ANOVA followed by the Tukey post-hoc test.

**
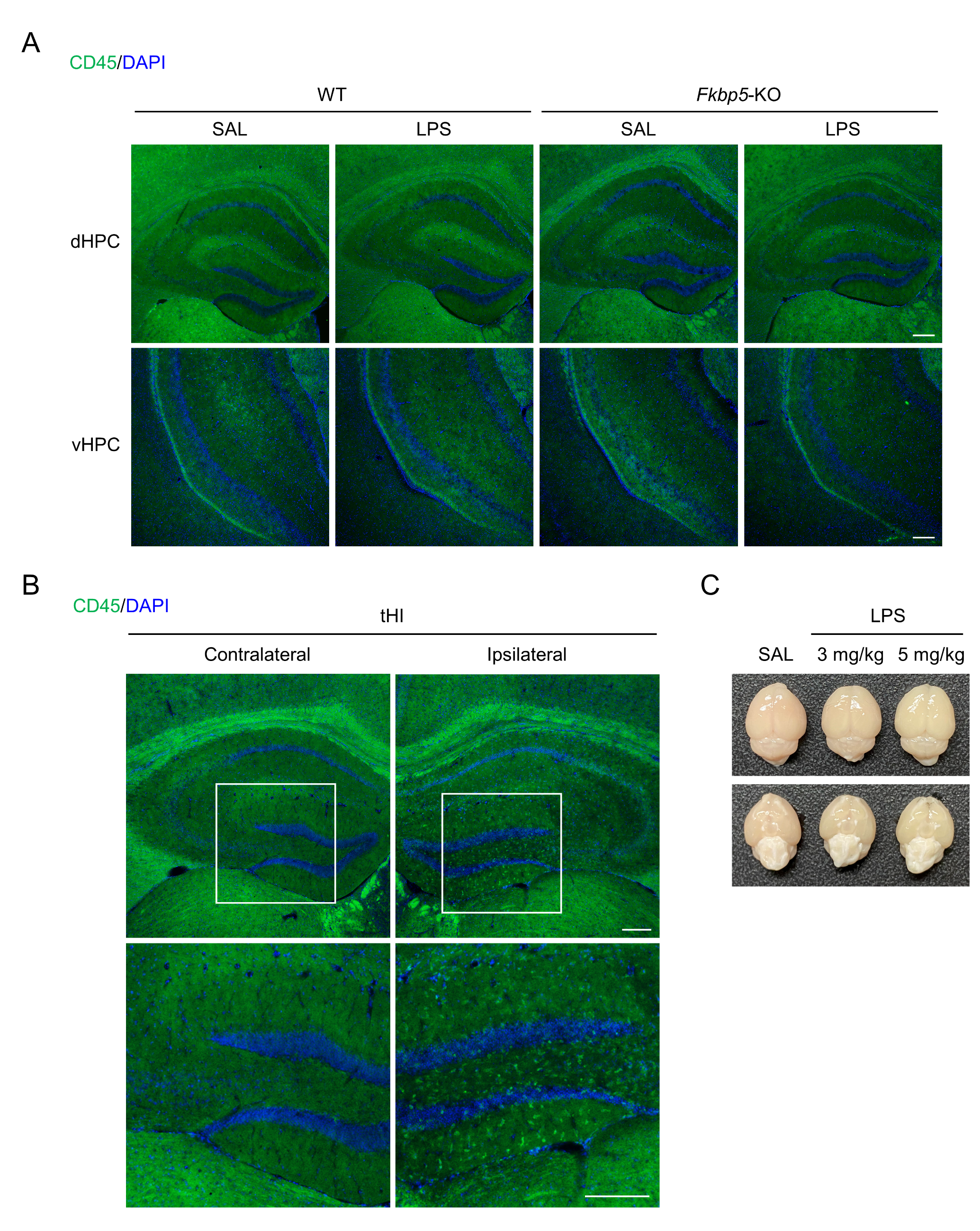
**

**Fig. S2 Peripheral injection of LPS did not induce the infiltration of leukocytes in hippocampus.**

**A** Representative immunofluorescence staining for leukocyte marker CD45 (green) and DAPI (blue) in the dorsal and ventral hippocampus 24 h after LPS injection. **B** The images of CD45 staining showed the leukocyte infiltration in hippocampus after transient hypoxia ischemic (tHI) stroke injury. Lower panel is zoom region of white square in upper panel. The ipsilateral hemisphere from tHI mouse brain served as a positive signal for CD45 staining. The CD45-positive cells were present in the hippocampus of ipsilateral hemisphere, but not in contralateral hemisphere. Scale bar: 200 μm. **C** The mice were intraperitoneally injected with 2% Evans blue dye 2 h after saline or LPS (3, 5 mg/kg of body weight) injection for analyzing the integrity of blood-brain barrier, and the brains were collected 4 h after injection of Evans blue. Representative images of brains displayed no Evans blue extravasation in all groups, suggesting that the LPS challenge used here did not induce detectable BBB leakage


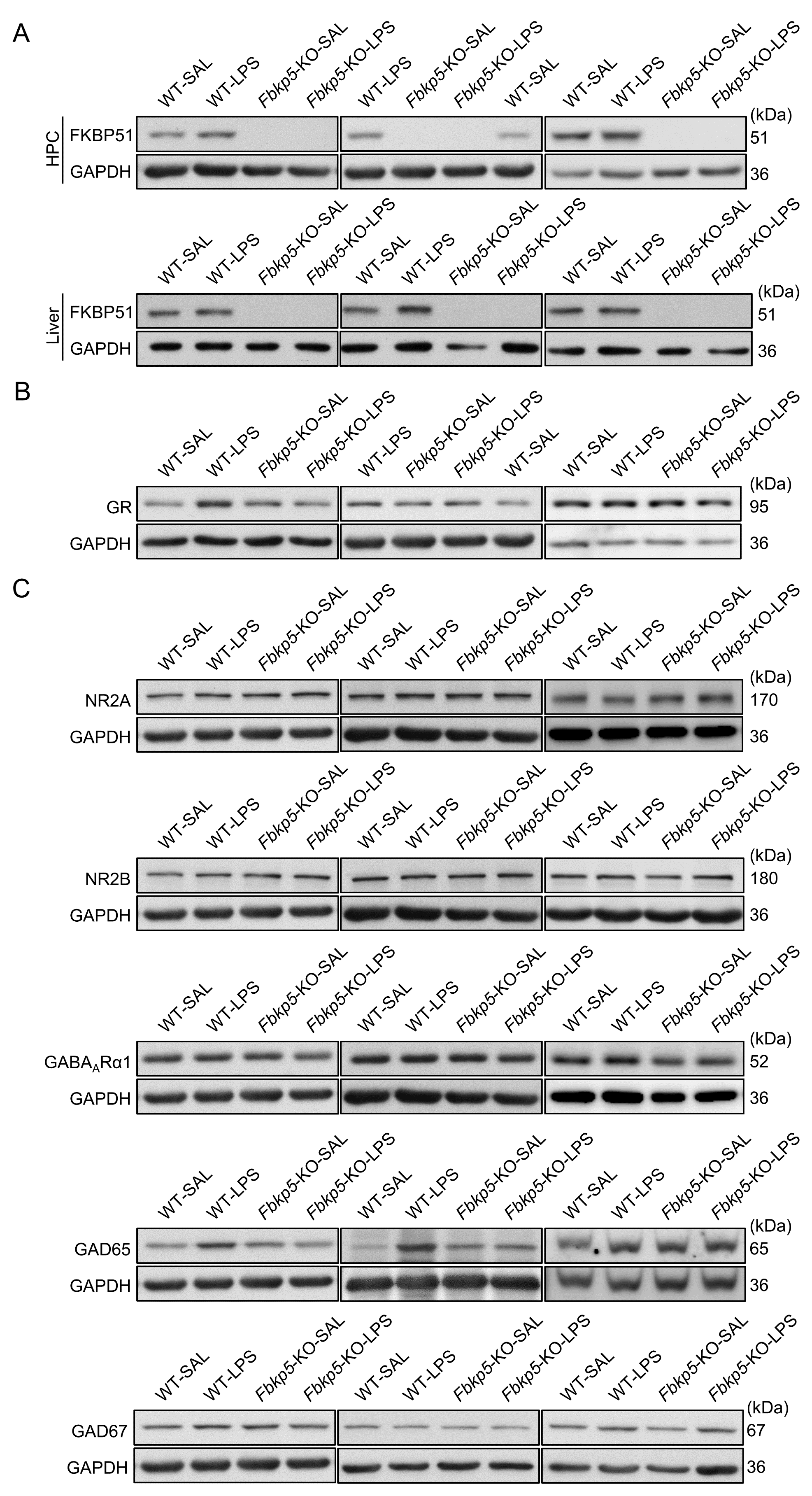


**Fig. S3** **Representative western blot used in quantification of protein expression in LPS model.**

The representative western blots from 3 animals per group related to Fig. 1L (A), Fig. 3C (B), Fig. 5A (C) in the main figures.


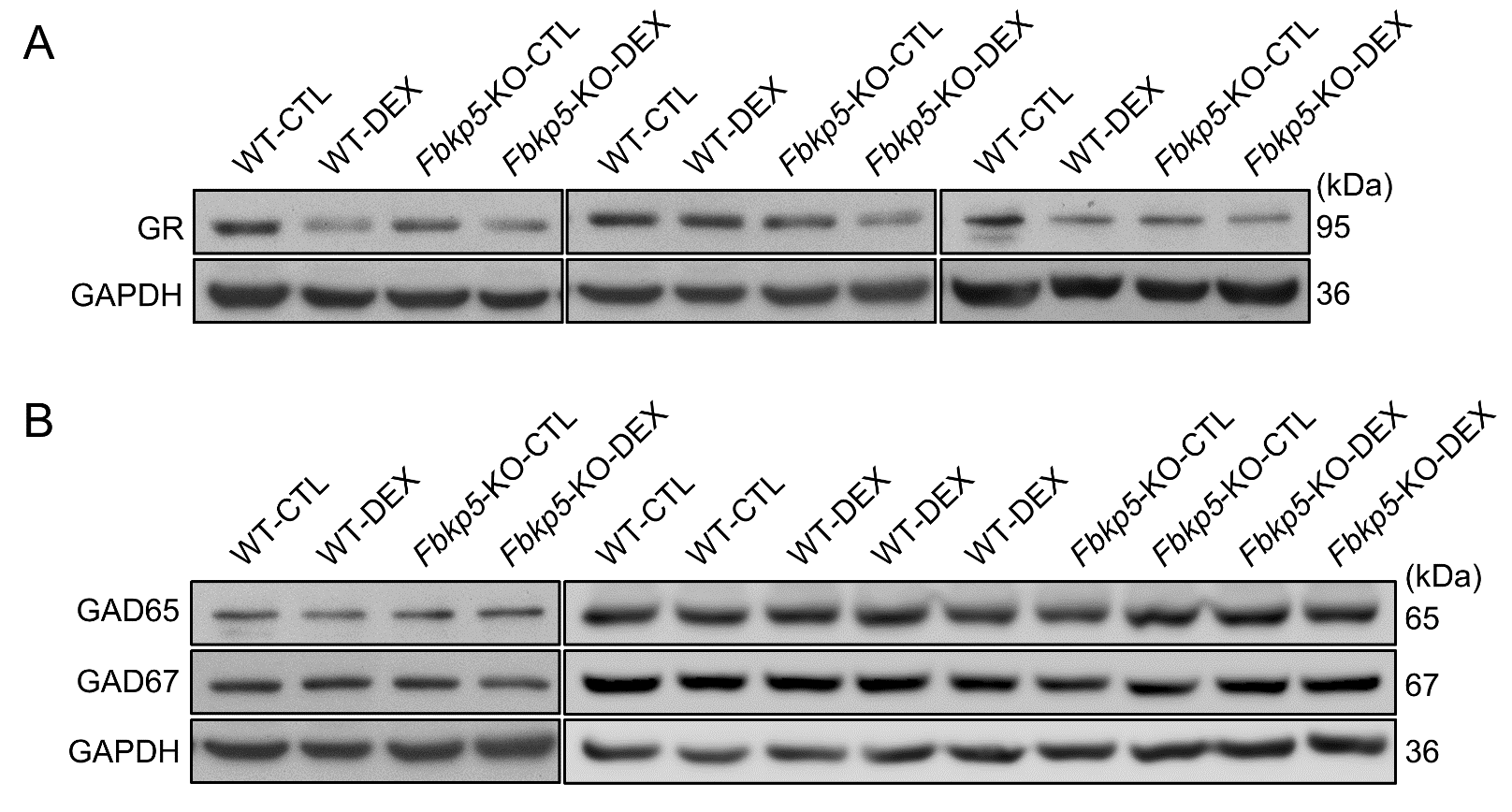


**Fig. S4** **Representative western blot used in quantification of protein expression in DEX model.**

The representative western blots from 3 to 4 animals per group related to Fig. 6H (A) and Fig. 6I (B) in the main figures.


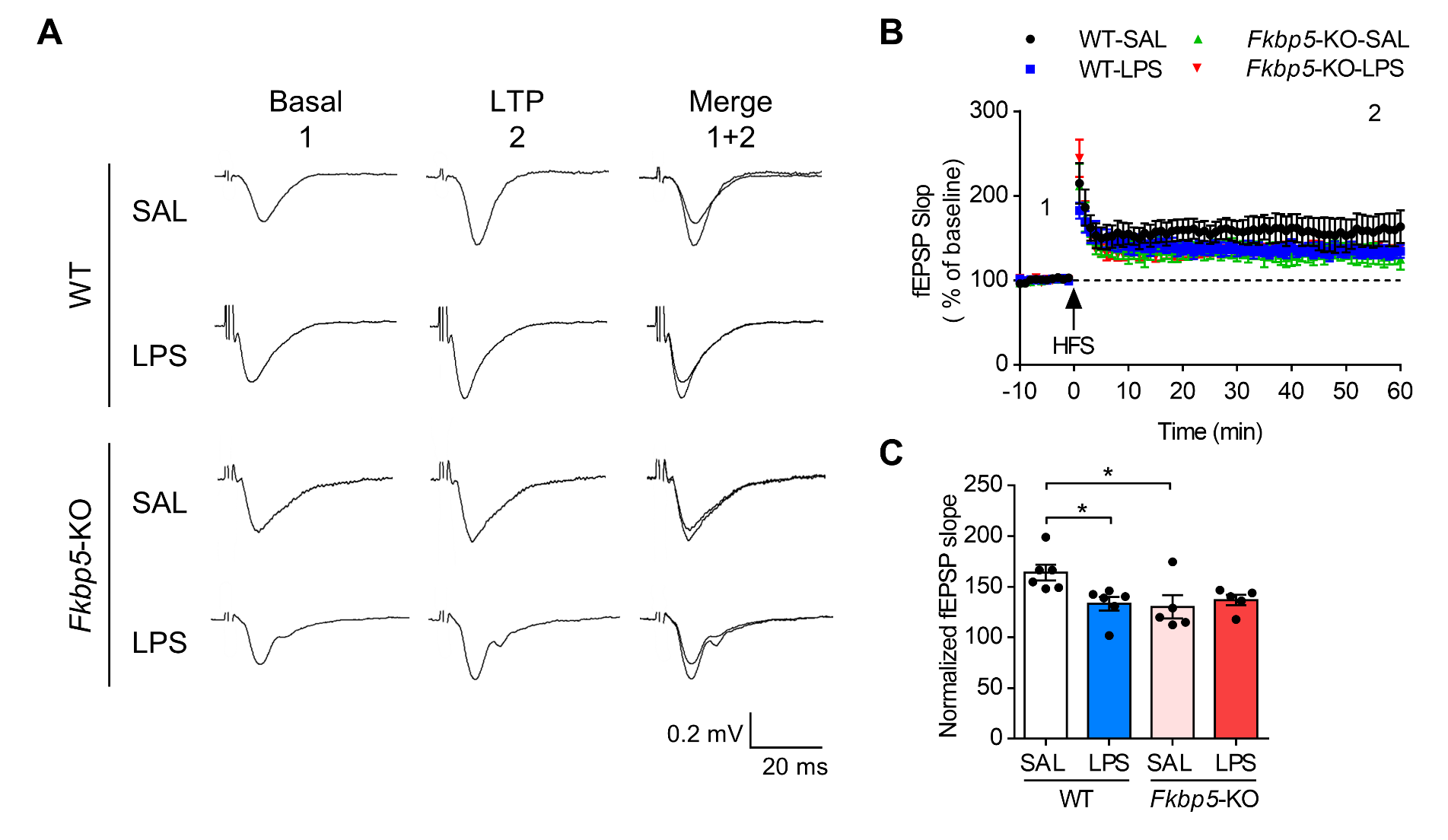


**Fig. S5 *Fkbp5* deficiency abolishes LPS-reduced LTP formation in dorsal CA1**

Mouse brain were harvested 7 days after the injection of SAL or LPS for high frequency stimulation (HFS)-induced long-term potentiation (LTP) measurement in the Schaffer collateral pathway. Dorsal hippocampal slices were stimulated by HFS to induce LTP. The field excitatory postsynaptic potential (fEPSP) was measure for 60 min following HFS and normalized to baseline. **A,** Representative trace. **B,** fEPSP slop. **C**, Normalized fEPSP slope. Data are expressed as the mean ± SEM (n = 5–6 mice per group). **p* < 0.05 for the comparison between two groups in a two-way ANOVA followed by the Tukey post-hoc test.


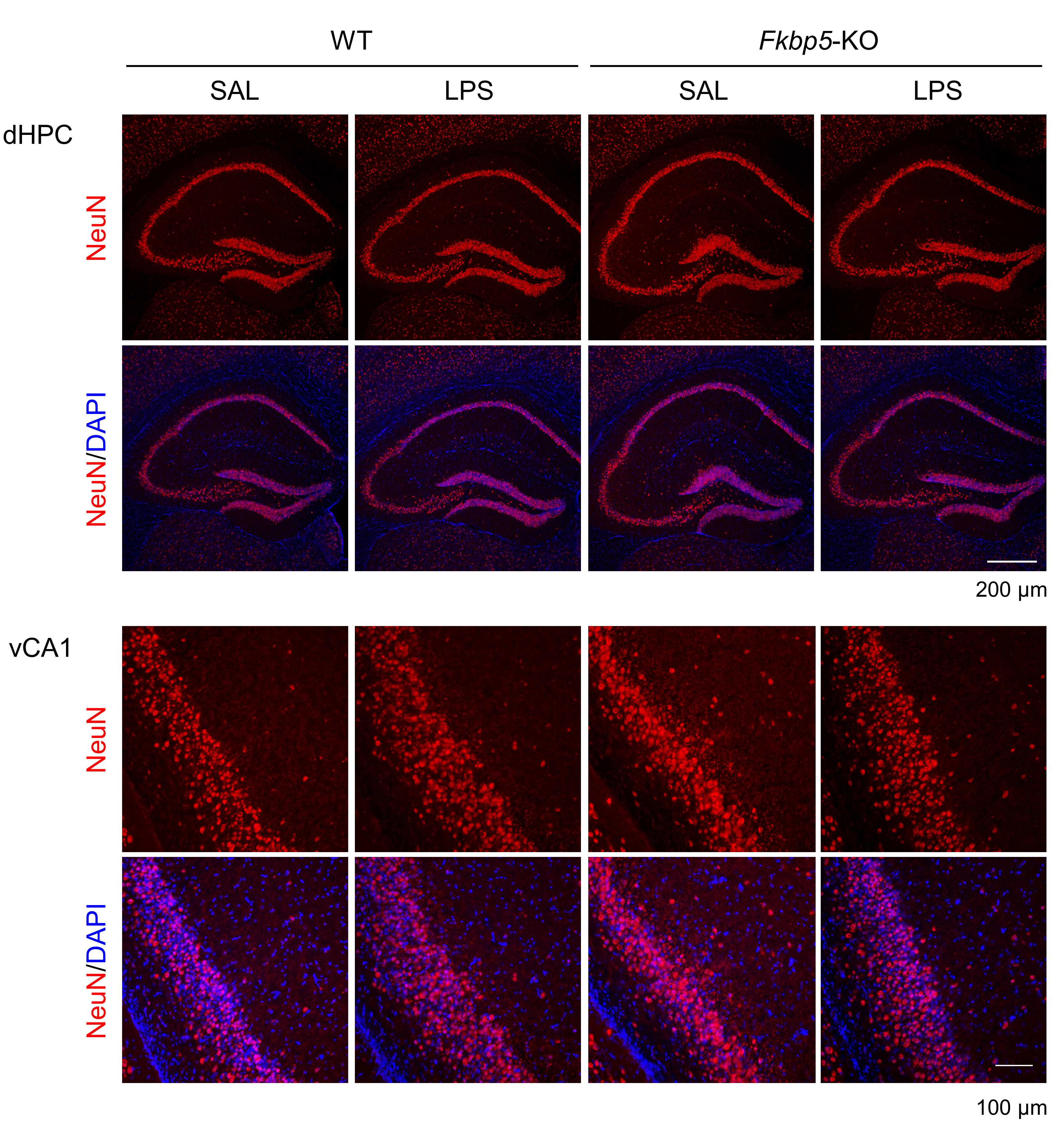


**Fig. S6 Peripheral injection of LPS did not affect neuronal cell viability in hippocampus.**

Representative immunofluorescence staining for neuronal marker NeuN (red) and DAPI (blue) in the dorsal hippocampus (upper plane, dHPC, scale bar: 200 μm) and ventral CA1 (lower plane, vCA1, scale bar: 100 μm) region of WT and *Fkbp5*-KO mice 7 day after SAL or LPS injection.

**Supplementary Materials and Methods**

***Transient Hypoxia Ischemic (tHI) Stroke Injury and leukocyte infiltration assessment***

Transient hypoxia ischemic stroke injury model was performed as previously described [1] with minor modifications. Briefly, twelve-week-old male mice were anesthetized under 2% isoflurane. We used two releasable knots of 4.0 silk suture to apply the transient occlusion of the right common carotid artery (CCA), and then exposed to 7.5% O_2_ / 92.5% N_2_ through a facemask for 25 min. After hypoxia, the ligation of CAA was removed. After 72 h, the mouse brain was collected for immunofluorescence staining analysis. Leukocyte infiltration was assessed by immunofluorescence staining of CD45 on mouse brain 7 days after LPS injection. The tHI mouse brain served as a positive signal of CD45 staining.

***Evaluation of Blood-Brain Barrier Permeability***

The mice were subjected with the intraperitoneal injection of saline or LPS (3 or 5 mg/kg of body weight) for 2 h, and then intraperitoneally injected with 2% Evans Blue dye in saline (4 mL/kg of body weight) for 4 h. Afterwards, the mice were transcardially perfused with ice-cold saline, the brain tissue was collected for analyzing the extravasation of Evans blue into mouse brain.

***Long-term potentiation (LTP) measurement***

Mice were sacrificed and sliced into 400-μm-thick coronal slices containing the hippocampal region. The brain slices were then maintained in a chamber containing artificial cerebral spinal fluid (aCSF; 117 mM NaCl, 4.7 mM KCl, 1.2 mM MgCl_2_, 1.2 mM NaH_2_PO_4_, 2.5 mM CaCl_2_, 25 mM NaHCO_3_, 11 mM glucose) solution saturated with 95% O_2_ and 5% CO_2_ stabled at room temperature for 1 hour before electrophysiological recordings. Evoked field potentials at 0.033 Hz were elicited by stimulation of Schaffer collateral/commissural pathway using a concentric bipolar stimulating electrode (FHC, Bowdoinham, ME, USA). A capillary glass recording electrode was positioned in the stratum radiatum of dorsal CA1 region to record the field excitatory postsynaptic potential (fEPSP). LTP was induced by three trains of high-frequency tetanic stimulus (HFS, 100 Hz, 1s with 1 min interval) and the fEPSP was monitored for an hour. The percentage of the mean fEPSP amplitude was increased following HFS.

**References**

1. Sun YY, Morozov YM, Yang D, Li Y, Dunn RS, Rakic P, Chan PH, Abe K, Lindquist DM, Kuan CY: **Synergy of combined tPA-edaravone therapy in experimental thrombotic stroke.** *PLoS One* 2014, **9:**e98807.
